# Joint Evolutionary Trees: A Large-Scale Method To Predict Protein Interfaces Based on Sequence Sampling

**DOI:** 10.1371/journal.pcbi.1000267

**Published:** 2009-01-23

**Authors:** Stefan Engelen, Ladislas A. Trojan, Sophie Sacquin-Mora, Richard Lavery, Alessandra Carbone

**Affiliations:** 1 Génomique Analytique, Université Pierre et Marie Curie-Paris 6, UMR S511, Paris, France; 2 INSERM, U511, Paris, France; 3 Laboratoire de Biochimie Théorique, IBPC, Paris, France; 4 Institut de Biologie et Chimie des Protéines, CNRS UMR 5086/IFR 128/Université de Lyon, Lyon, France; Stanford University, United States of America

## Abstract

The Joint Evolutionary Trees (JET) method detects protein interfaces, the core residues involved in the folding process, and residues susceptible to site-directed mutagenesis and relevant to molecular recognition. The approach, based on the Evolutionary Trace (ET) method, introduces a novel way to treat evolutionary information. Families of homologous sequences are analyzed through a Gibbs-like sampling of distance trees to reduce effects of erroneous multiple alignment and impacts of weakly homologous sequences on distance tree construction. The sampling method makes sequence analysis more sensitive to functional and structural importance of individual residues by avoiding effects of the overrepresentation of highly homologous sequences and improves computational efficiency. A carefully designed clustering method is parametrized on the target structure to detect and extend patches on protein surfaces into predicted interaction sites. Clustering takes into account residues' physical-chemical properties as well as conservation. Large-scale application of JET requires the system to be adjustable for different datasets and to guarantee predictions even if the signal is low. Flexibility was achieved by a careful treatment of the number of retrieved sequences, the amino acid distance between sequences, and the selective thresholds for cluster identification. An iterative version of JET (iJET) that guarantees finding the most likely interface residues is proposed as the appropriate tool for large-scale predictions. Tests are carried out on the Huang database of 62 heterodimer, homodimer, and transient complexes and on 265 interfaces belonging to signal transduction proteins, enzymes, inhibitors, antibodies, antigens, and others. A specific set of proteins chosen for their special functional and structural properties illustrate JET behavior on a large variety of interactions covering proteins, ligands, DNA, and RNA. JET is compared at a large scale to ET and to Consurf, Rate4Site, siteFiNDER|3D, and SCORECONS on specific structures. A significant improvement in performance and computational efficiency is shown.

## Author Summary

Information obtained on the structure of macromolecular complexes is important for identifying functionally important partners but also for determining how such interactions will be perturbed by natural or engineered site mutations. Hence, to fully understand or control biological processes we need to predict in the most accurate manner protein interfaces for a protein structure, possibly without knowing its partners. Joint Evolutionary Trees (JET) is a method designed to detect very different types of interactions of a protein with another protein, ligands, DNA, and RNA. It uses a carefully designed sampling method, making sequence analysis more sensitive to the functional and structural importance of individual residues, and a clustering method parametrized on the target structure for the detection of patches on protein surfaces and their extension into predicted interaction sites. JET is a large-scale method, highly accurate and potentially applicable to search for protein partners.

## Introduction

Interface residues are essential for understanding interaction mechanisms and are often potential drug targets. Reliable identification of residues that belong to a protein-protein interface typically requires information on protein structures [1] and knowledge of both partners. Unfortunately, this information is often unavailable and for this reason, reliable site prediction using a single protein, independently from its partners, becomes particularly valuable. Interactions of a protein with ligands, other proteins, DNA or RNA are all characterized by sites which either are conserved, present specific physical-chemical properties or fit a given geometrical shape [2],[3]. At times, the interface presents a mixture of these three signals.

Interfaces differ from the rest of the protein surface typically because buried interface residues are more conserved than partially buried ones and because the sequences associated with interfaces have undergone few insertions or deletions. However, on average, the most conserved patches of residues overlap only the 37.5% (±28%) of the actual protein interface and an analysis of 64 different types of protein interfaces (formed from close homologs/orthologs or from diverse homologs/paralogs) demonstrated that conserved patches cannot clearly discriminate protein interfaces [4].

The composition of interacting residues appears to distinguish between different types of interfaces [5],[6]. In particular, hydrophobic residues [7] and specific charge distributions [5],[8] have been shown to be characteristic of protein-protein interfaces. Protein interaction sites with ligands, DNA and RNA are usually highly conserved and the signal of conservation is likely to be sufficient for good predictions. The same does not hold true for protein-protein interfaces, where we show that combining information coming from conservation and the specific physical-chemical properties of the interacting residues, enhances the signal.

We propose a predictive method, named Joint Evolutionary Trees (JET), that extracts the level of conservation of each protein residue from evolutionary information, combines this information with specific physical-chemical properties of the residues, and predicts conserved patches on the protein surface of known three-dimensional structures. Defined in this way, JET is able to detect protein interfaces with very different types. It does not require information on potential interaction partners and it belongs to the family of methods which have been inspired by the Evolutionary Trace approach (ET) [9],[10]. Similarly to ET, JET analyzes a protein sequence 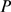 and structure, and finds information (from a careful analysis of the evolutionary distances between sequences homologous to 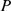) on binding interfaces by detecting conserved patches on the surface of the structure of 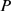. JET has been designed with large-scale applications in mind which requires the approach to be adjustable for different datasets and to guarantee predictions even with weak signals. Because of this, various evolutionary hypotheses on protein interfaces have been tested and new methodological approaches have been developed within JET.

Two main hypothesis on interaction sites have been tested. The first asserts that specific physical-chemical properties of patches always co-exist with some degree of conservation of the patch. The second claims that interaction sites on a protein surface are composed of an internal core which is conserved, with concentric layers of residues around the core which are progressively less conserved.

We also addressed four main methodological points. The first concerns the problem of accurately quantifying the strength of residue conservation in a set of sequences whose similarity to 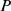 has been automatically evaluated by PSI-BLAST. This means reducing the interfering effects of sequences wrongly selected by PSI-BLAST (that is, sequences that are not homologous to 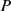) on the topology of the associated distance tree, and ensuring, as far as possible, diverse sequence identity within the samples. To this end, we introduce a new discrete combinatorial paradigm of computation to investigate potentially large sets of biological sequences by randomly sampling small subsets a sufficient number of times to ensure statistical overlap of the sampled sets. This method turns out to be powerful and also computationally efficient.

The second point concerns the core of the ET methodology which relies on the definition of a *trace*, a notion that quantifies the conservation of a residue position within a distance tree of sequences similar to 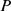 and that was originally introduced in [9]. This definition turns out to be insufficient to properly characterize residue conservation and a “hybrid” definition was proposed in [11] which combines the original notion of a trace, based on tree topology, with information entropy of the residue position within the pool of aligned sequences. In JET, we clarify the limits of the original combinatorial definition by redefining a trace based on tree topology and demonstrate that information entropy is not required.

The third point concerns the evaluation of patches of conserved residues as potential internal cores of interaction sites. We tested the hypothesis that such cores correspond to the largest patches found for the protein and observed that this is generally the case. A novel method estimating the size of relevant clusters of conserved residues and of clusters of residues with specific physical-chemical properties has been tailored around the specific protein being treated 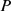. The method is based on a random generation of clusters over the protein surface of the protein in question 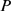. An evaluation of the size of a cluster based on a random generation is used also in [11]. The important difference between the two approaches is that, in the latter case, the estimation is made for arbitrary proteins.

Finally, since JET is based on the random choice of small sets of sequences for constructing multiple trees, it could yield slightly different answers in different runs. This fluctuation has been analyzed and exploited to further improve our algorithm. An iterative version of JET (iJET) provides a list of consensus residues belonging to interaction patches. When JET is used for large-scale analyses, this turns out to be a safe and successful approach. When the user uses JET on a single protein, it is possible to run it once, or to explore the set of potentially interacting residues by varying a consensus threshold during iterations. In difficult cases, this can allow the user to refine the detection of interacting residues.

## Materials and Methods

The sequence 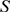 corresponding to the available PDB structure is called *reference sequence*.

Below, we describe in detail the basic steps constituting JET. The methods developed for each step are designed for large-scale applications. The aim is to insure that the system always provides a prediction even with weak signals. To achieve this we made the approach adaptable to different datasets in terms of the number of retrieved sequences, the amino acid distance between sequences, and the selective thresholds for cluster identification.

JET first recovers a set of sequences homologous to 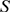 using PSI-BLAST and selects a pool of sequences that uniformly represents a broad range of sequence identities. These sequences are then used to construct a large number of small distance trees that will be analyzed to determine the importance of the residues in 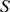. Based on the residue ranking JET clusters together the most important residues and detects patches on the surface of the three-dimensional structure, predicted to be potential binding sites.

### PSI-BLAST Search

JET performs a PSI-BLAST search [12] at http://www.ncbi.nlm.nih.gov/Blast.cgi, or locally, to select as many as 5000 sequences. It does it on chains with at least 20 residues. Retrieved sequences are filtered to eliminate redundant sequences, that is sequences with >98% sequence identity to 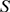, and to eliminate very divergent sequences, that is sequences with <20% sequence identity.

A second filter is defined on the length of the alignment which should cover at least the 80% of the length of the reference sequence 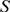, and on the number of inserted gaps which should be <10% of the size of the alignment.

A third filter cuts-off sequences with an e-value ≥10^−5^.

If the pool of remaining sequences does not contain at least 100 sequences, then the cut-off on the length of retrieved alignments is automatically decreased by 10% of the length of 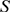 progressively until reaching 51% of the length of the reference sequence (this condition ensures that all selected sequences will overlap with each other). If the number of sequences retrieved is insufficient, we reset the length of the alignment to 80% of the length of the reference sequence 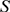 and restart the analysis with an e-value of 10^−4^. We repeatedly increase the e-value and decrease the length by filtering sequences progressively with e-values 10^−3^, 10^−2^, 10^−1^, 1, 10, 100, until a sufficient number of sequences is retrieved.

At the end of the retrieval step we obtain a set 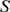 of selected sequences.

### Gibbs-like Sampling of Sequences Chosen with PSI-BLAST

We want to align small sets of 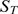 sequences in 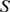 approximately 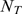 times. With the purpose of using most of the information contained in 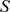 and to guarantee overlapping of sequences among trees, we set 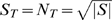 whenever 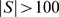 and we fix 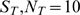 otherwise. Each set of 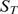 sequences contains the reference sequence 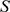. Since the distribution of sequences based on sequence identity might not be uniform, we order sequences in 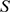 in four classes characterized by 20–39% (including 20 and 39), 40–59%, 60–79%, and 80–98% sequence identity. This ensures a comparable set of representatives for different groups of identity within each set of aligned sequences. We then randomly select 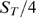 distinct sequences from each class. (If 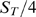 is not an integer, we pick the remaining sequences, that is 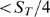 sequences, successively, starting from the class of sequences characterized by the smallest sequence identity.) We require that each class contains enough sequences to ensure diversity within the 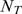 generated alignments. Ideally, this corresponds to requiring that the inequality 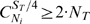 holds, where 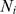 is the number of distinct sequences in the 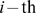 class with 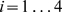. In practice, we may find classes with insufficiently varied sequences to supply the 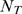 sets to be aligned. In this case, if the class is empty, we ignore it. If it is not empty, we decrease the number of sequences to pick up within this class to a maximum 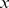 such that 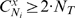. We pick the missing 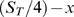 sequences from the other classes, satisfying 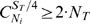. We order the classes with respect to the combinations 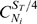 and choose the sequences starting from the class with greatest value. In the event that there is a class where the inequality cannot be satisfied due to lack of sequences, we decrease the coefficient 2 within the inequalities (for all 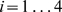) by a maximum of five steps towards the coefficient 1. For each step we apply the procedure above to the new class of inequalities.

This way, we obtain a good compromise between an ideally uniform distribution of sequence identities within an alignment and the diversity of sequences amongst different alignments.

### Multiple Sequence Alignments and Trees Construction

Sequences in a pool are aligned using CLUSTALW with the Blosum62 matrix [13]. The Score Distance method [14] has been used to define the distances between sequences obtained by the alignment; no contribution is made for gaps in the sequence nor by the ends.

To align distantly related proteins, Gonnet [15] and HSDM [16] matrices are preferable and an automatic selection between Blosum62, Gonnet and HSDM has been implemented in JET. The criteria is as follows. Given an alignment of two sequences 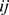 the score distance method computes the effective score of the alignment
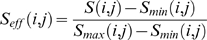
where 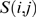 is the score produced by the alignment using a substitution matrix, 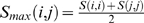, 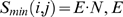 is the e-score value of the matrix (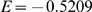 for Blosum62, 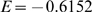 for Gonnet, and 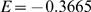 for HSDM) and N is the number of pairs of aligned residues 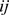. Based on this, one computes distances between two sequences as 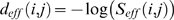 To properly compute distances, one needs to guarantee 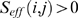. In the case of distantly related proteins, it is possible that 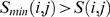 and the value can become negative. When this occurs for some pairs 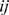 using Blosum62, we take sequences 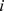 and 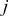 (whenever different from the reference sequence 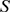) out of the set and recompute distances until the condition is satisfied for all pairs. We require that the number of sequences in the tree covers 75% of the original number of sequences and is ≥10 (this corresponds to the minimal size of an acceptable tree). If at least one of these conditions is not satisfied then we repeat the analysis using the Gonnet method. If this also fails to pass the test the HSDM method will be used.

For each multiple alignment, a distance tree is constructed based on the Neighbor Joining algorithm (NJ) [17]. The midpoint rooting method is used to find the point that is equidistant from the two farthest points of the tree, and to root the tree there.

### Tree Analysis and Tree Traces

**Figure 1:**
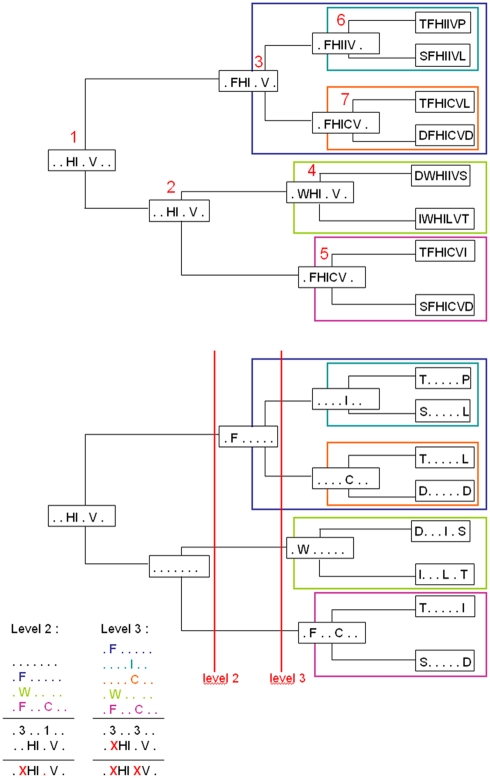
Schema of the tree trace computation. Top: tree with nodes labeled with consensus sequences: conserved residues are traced from the leaves back to the root. Ranks of nodes are labeled in red and 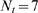. Subtrees of nodes of rank 2 and 3 are contoured with colored boxes. Bottom: tree with nodes labeled with back-trace sequences: back-traces are traced from the root back to the leaves. 3 subtrees corresponding to level 2 (blue, green and rose boxes) and 4 to level 3 (turquoise, orange, green and rose boxes). On the bottom left, schema of the computation of tree traces of level 2 and 3 based on 3 and 4 subtrees. Tree traces of level 2 (3) occupies the second (fifth) position in the sequence and it is denoted by X.

If 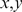 are two nodes belonging to a branch of 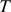, let 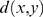 be the distance between 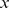 and 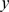 provided by the tree construction. The root of 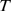 has rank 1. A node 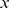, which is not a leaf, has *rank* 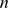, if all nodes 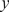 of 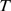 such that 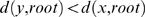 have rank 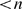 and at least one of them has rank 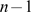. If two nodes 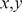 (which are not leaves) are such that 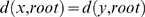 then their rank is the same. The maximum rank definable on a tree 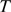 is 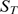, that is the number of sequences in 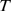 See Figure 1, top.

*Consensus sequences of rank n* and *backtrace sequences of rank n* are used to define *tree traces*.

Let 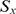 be the sequence associated with the leaf 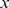 in 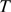. A *consensus sequence associated to a leaf* 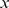 of 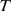 is a sequence (of the same length as 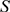) where position 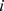 is occupied by the residue in 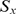 aligned to the *i*-th residue of 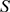 If no residue in 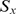 is aligned to the *i*-th position of 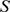 then a gap will appear in the consensus sequence. A *consensus sequence of a node* 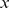 *of rank n* is a sequence (of the same length as 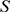) where the 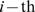 position is occupied by those residues common to the consensus sequences associated with the children of 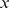. See Figure 1, top.

A *back-trace sequence of a node* 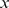 *of rank* 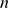, is a sequence (of the same length as 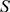) which records all residues in the consensus sequence associated to 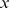 that do not already belong to the back-trace of the father of 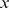. The back-trace sequence of the root is the consensus sequence of the root. See Figure 1, bottom.

Given 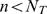, let 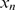 be a node in 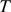 with rank 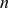; we look at all positions 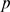 along the branches of 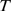 such that 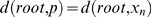 and we collect in a set 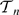 subtrees of 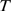 associated with positions of level 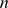 as follows: given a position of level 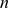 along some branch (defined below), we include the subtree of 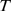 rooted at this point in 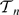 only if the subtree contains more than two nodes; if the position coincides with a branching node of 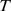, then we include two copies of the subtree in 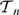. Each subtree in 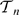 has a backtrace associated to its root. A *tree trace of level* 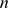 is a residue which is not a tree trace of level  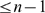 and that occurs in backtraces of at least 2 subtrees in 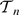. A residue in the backtrace sequence of the root of 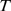 is conserved in all sequences, in particular in 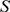, and it is called a *tree trace of level* 1.

**Figure 2:**
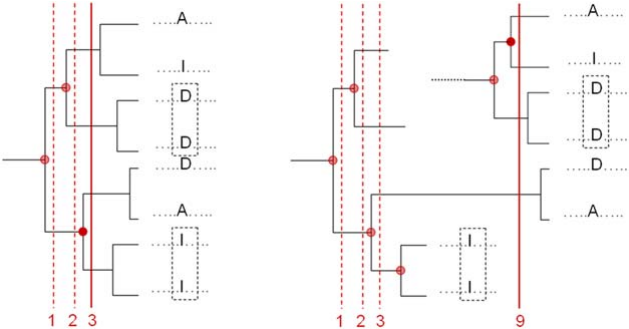
Examples of tree trace levels. Left: residues I and D at position 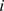 in the alignment are conserved in two subtrees (dotted box), and this sets 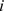 as a tree trace of level 3. Right: residue I and D are conserved in two subtrees detectable at levels 3 and 9 respectively, and this sets 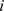 as a tree trace of level 9.

Notice that this definition is much weaker than the corresponding definition of trace for ET. In fact, in ET, a residue is a trace of level 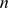 only when the residue is conserved in *all* subtrees of 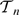. See Figure 2.

### Relative Trace Significance and Average Trace Value

The set of tree traces resulting from the analysis of all generated metric trees will be used to define the *relative trace significance* for the residues in the PDB structure. Let 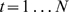 be the generated trees, and 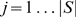 index the residue positions in 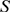. We say that a residue 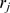 at position 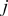 in 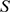 is a trace with degree of significance
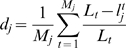
where 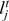 is the tree trace level of residue 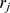 in tree 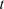, 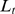 is the maximum level of 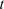 and 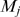 is the number of trees where the residue appears as a trace. Values 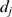 vary in the interval [0,1], and represent an average over all trees of the residue importance: traces appearing often at small (big) levels will get values close to 1 (0). We can consider 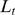 in the formula to be smaller than the maximum level attainable, that is 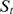. This corresponds to the 95% (a default parameter of the method) of residues which have a trace value for a tree. Note that the condition does not imply that some residues have no trace (indeed traces are read out of many trees).

The *average trace value* for a residue 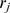 is computed with respect to the relative trace significance of it and the one of its neighboring residues:
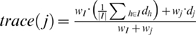
where 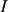 is the set of residue positions which are neighbors of 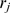 (that is, a neighbor is a residue with a distance <5*Å* from 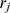 of at least one of its atoms), and where we fixed by default the weight values at 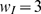 and 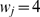, favoring the residue 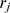 compared to its neighbors. 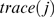 is the actual value that is used in JET to rank residues and to establish the importance of a residue position 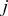.

### Surface Atoms, Surface Residues, and Surface Clusters

**Figure 3:**
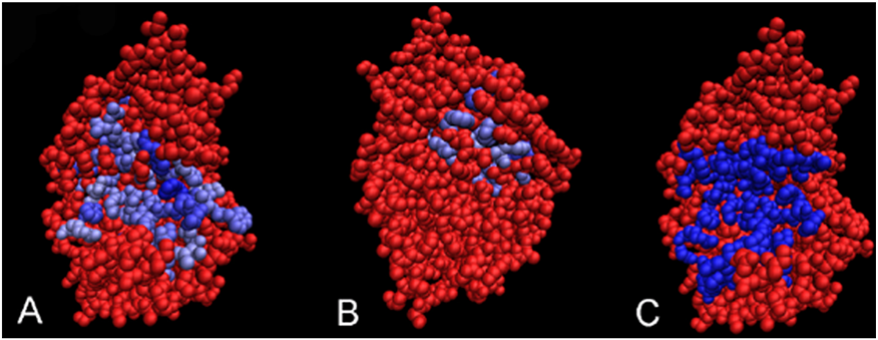
Clustering based on surface atoms. Structure of the catalytic subunit of cAMP-dependent protein kinase (PDB file 1apm). The experimental interaction site is colored blue in (C). Clustering based on surface residues detects one conserved cluster that gives rise to two non-contiguous surface patches. One of them (A) corresponds to the actual interface and the other (B), which is positioned opposite to (A), does not. Clustering based on surface atoms distinguishes the two patches and considers only one of them (A) as a cluster seed.

*Surface residues* are residues with at least 5% of accessible surface [18]. *Surface atoms* have at least 1Å^2^ of accessible surface. Accessibility is calculated with NACCESS 2.1.1 [19] with a probe size of = 1.4Å. In practice we shall use surface atoms belonging to surface residues only. A *surface cluster* is a set of surface residues to which a residue 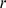 belongs if at least one of the surface atoms of 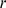 is at distance <5Å from a surface atom in some other residue of the cluster. Several surface clusters can be detected for a single protein. Note that a surface cluster contains residues that are in contact because of surface atoms and excludes contacts based on internal atoms. As a consequence of this definition, clusters which are not contiguous patches at the protein surface are separated and, in some cases, several smaller surface clusters are obtained. See Figure 3.

This definition reflects the idea that protein-protein interactions depend on atomic-level detail.

### Number of Residues on Protein Surfaces and Average Interface Fractions

**Figure 4:**
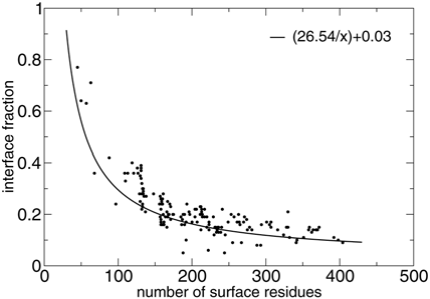
Protein surface size and interface fraction. Plot of the 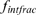 function relating surface size and fraction of the surface covered by the interface. Dots correspond to JET predictions on all proteins in the Huang dataset, where predicted interface sites are computed with mixed trace values.

An inverse relation between the fraction of the surface covered by the interface and the total protein surface has been observed in [20] based on a dataset of 1256 protein chains. We approximated the data in [20] with the function 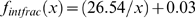 (plotted in Figure 4), where 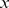 is the number of surface residues. We used this analytical expression to parameterize the clustering algorithm described below.

### Clustering Algorithm with Seeds

Two thresholds are defined from the distribution of trace values computed with JET. The *cluster-trace threshold* is the trace determined with a confidence level of 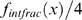 on the distribution of trace values and the *residue-trace threshold* is the trace determined with a confidence level of 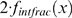 for the same distribution. These thresholds are used to construct and evaluate appropriate clusters.

The clustering algorithm is structured in three steps. The first two steps are used to construct “cluster seeds” that will be extended into clusters at the third step of the construction.

First, the algorithm orders all trace residues from the largest to the smallest. Next, it chooses residues with the highest trace value, greater than the residue-trace threshold, and either creates a new isolated cluster or adds the residue to an old cluster by checking that the average trace of the new cluster (either the isolated one or the one obtained by extension) is greater than the cluster-trace threshold. Notice that residue traces may be smaller than the cluster trace threshold. The set of clusters 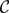 obtained in this way is filtered by the next step of the algorithm.

In the second step, the algorithm computes a threshold for the size of the “cluster seeds”. To do so it takes the distribution of trace values obtained by running JET on a given protein and randomly reassigns the same trace distribution to surface residues of the protein. It clusters with the clustering algorithm described above and repeats this procedure 6000 times. It calculates the distribution of the size of the clusters and the distribution of the number of clusters obtained, to determine the *percentile of a size* 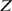, that is, the fraction of the population which has a size 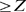, and the *percentile of the number of clusters* 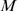, that is the fraction of the population with a number of clusters 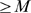.

Then it selects the clusters in 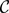 (obtained in the first step) those within a percentile of size <0.1. For all other clusters 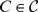, it considers more relaxed conditions for selection. Namely, it selects clusters 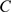 which are smaller in size, but have a high average trace compared to the others in 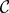. This notion is coded into the following two numerical conditions:
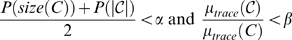
where 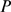 computes the percentile in a distribution and 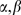 are set at 0.15 and 1, respectively, for a first round of selection and to 0.25 and 0.95 for a second round of selection. If no cluster is selected, then the algorithm goes back to the random distribution, repeats the analysis by increasing the percentile level by 10% and recomputes a new, more lax, threshold until at least one cluster is found. The clusters obtained at the end of the second step of the clustering algorithm are called *cluster seeds*.

The third step of the algorithm extends the cluster seeds with neighboring residues by maintaining a sufficiently high average trace of the cluster. To do this, the cluster-trace threshold is set at a confidence level of 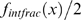. Neighboring surface residues are those that respect the definition of a cluster once added. The algorithm collects all neighboring surface residues and adds them one by one by decreasing trace value, each time checking that the cluster-trace and the residue-trace thresholds are respected. When all neighboring residues are treated, the algorithm extends the resulting cluster further by searching for a new set of neighboring surface residues and by applying the extension procedure described before until no further extension is obtained.

The algorithm then outputs the final set of clusters. The number of clusters may be smaller than the number of cluster seeds because extension may lead to the fusion of some initial clusters.

Note that the residue-trace threshold guarantees that we are going to cluster a pool of residues among the 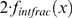 best trace residues. The cluster-trace is used to guarantee that the average trace of the seed cluster remains high.

### Detection of Sites Based on Physical-Chemical Signals

Statistical analysis of physical-chemical properties of protein-protein interfaces reveals a biased amino-acid content within interfaces and allows the definition of propensity values for interface residues [21]. These values are listed in Text S1. We use propensity values to rank residues in a protein. For each residue 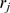 we define
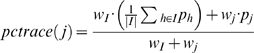
where 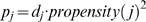, 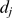 is the degree of significance of 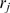, 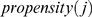 is the propensity value of 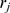. Notice that the formula is similar to 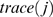 and parameters 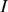, 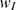 and 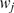 are defined as for 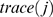. We employ the ranking on 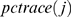 for computing cluster seeds 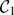 based on physical-chemical signals by running the first and second step of the clustering procedure (with a cluster-trace threshold determined by using the distribution of trace values dependent on 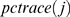). Then we compute cluster seeds 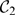 based on conservation using the ranking of trace values (with a cluster-trace threshold computed from the trace distribution). Cluster seeds in the set 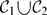 are extended with the third step of the clustering procedure. For this we use a *mixed trace* value for a residue 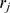 at position 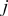, instead of the usual trace value, which is defined as
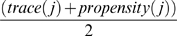
that is, the average between trace and propensity. The cluster-trace threshold is computed from the distribution of mixed trace values. Note that cluster seeds detected by different signals can again fuse into a single cluster as discussed later for a allophycocyanin structure.

Size of predicted clusters computed with mixed trace values and number of surface residues are reported in Figure 4 for all proteins in the Huang dataset. Points fluctuate around the 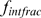 curve (which represents reference values) and this is due to the multiple parameters used for clusterisation.

### Automatic Determination of Experimental Interaction Sites from Known Complexes

The experimental interaction sites for the proteins listed in Text S2, Text S3 and Text S5 are determined using the crystal structure of the protein and NACCESS [19] for the detection of residues exposed to the solvent.

**Figure 5:**
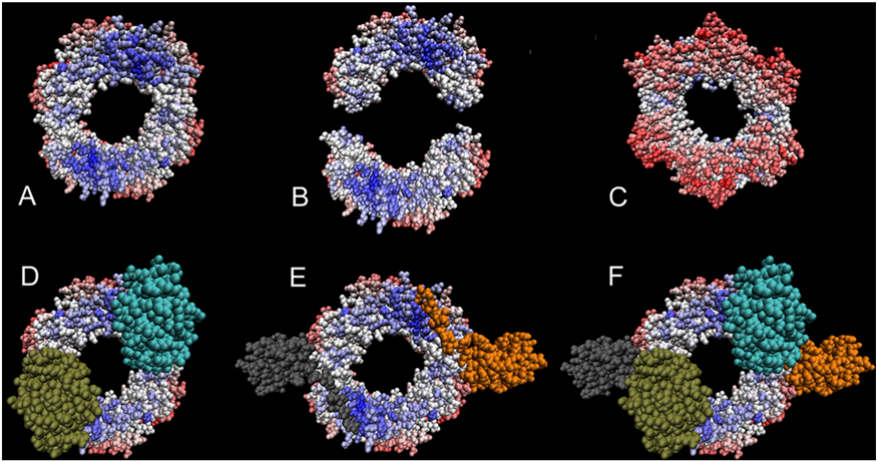
JET prediction of multiple interaction sites based on conservation signals, and known binding complexes. 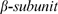
 of *Escherichia coli* DNA polymerase III holoenzyme; the PDB file 2pol contains the two homodimeric chains. Top: Complex shown in (A) and (C) is formed of two monomers which are shown slightly separated and inclined away from the viewer in (B). Conserved residues are colored from blue (most highly conserved) to white, and non-conserved residues are colored from white to red (no trace of conservation being found). Note the conserved zones at the contact surfaces between the two monomers (visible for the upper monomer in (B)). The complex shows a conserved face (A) and a non-conserved one (C). Bottom: the conserved face is in contact with two other chains (PDB files 1jql:B (D) and 1unn:CD (E)) which are not included in the PDB file 2pol. The results of JET can be understood when chains 1jql:B and 1unn:CD are added to 2pol giving meaning to the conserved sites detected.

JET finds signals corresponding to different interactions of a protein, namely with other proteins, ligands, DNA or RNA, as well as the chain-chain interactions in multimeric proteins. Hence, it becomes important to consider all it is known of such interactions to correctly evaluate predictions (see Figure 5). Given a protein, we considered all interactions between its chains. In addition, we collected information on other potential interactions by searching in the PDB archive for protein complexes containing a chain that displays at least 95% sequence identity to a chain in the PDB file of the experimental structure. All sites for the homologous chains (defined by an interaction with other chains in the “homologous” PDB file) are considered. For all PDB files (the reference and the homologous ones), we also looked at all chain-ligand interactions described in them, and selected those involving ligands that are known to have a functional role. For this, we used a list of enzyme compounds associated to reactions stored in KEGG database (a flatfile was downloaded at ftp://ftp.genome.jp/pub/kegg/ligand/enzyme/enzyme) and discharged all compounds which were absent in the list. All identified interactions were grouped together to define the set of “true” interacting residues of the experimental structure to be evaluated. We define a residue to belong to an interaction site if at least 10% of the accessible surface of the residue (within the protein) becomes non accessible due to the interaction (within the complex).

### Evaluation of JET

To properly evaluate JET performance on a given protein we rely on the following quantities: the number of residues correctly predicted as interacting (true positives, TP), the number of residues correctly predicted as non-interacting (true negatives, TN), the number of non-interacting residues incorrectly predicted as interacting (false positives, FP) and the number of interacting residues incorrectly predicted as non-interacting (false negatives, FN). We use four standard measures of performance: sensitivity 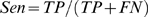, specificity 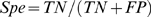 accuracy 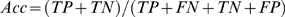 and positive predictive value 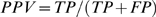. We also consider scores to evaluate the statistical pertinence of the above measures. Expected values are calculated as 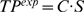, 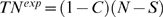, 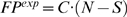, 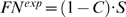, where 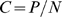 is the coverage obtained with JET, 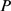 is the number of surface residues predicted by JET, 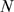 is the total number of surface residues and 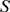 is the number of residues in the real interaction site. Note that the calculation of expected values assumes that 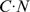 residues have been selected at random as being positives on the structure of the protein under study. This means that expected values depend on the protein studied. We can now compute sensitivity 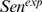, specificity 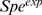 accuracy 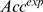 and positive predictive values 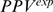 for the random case: 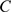, 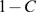, 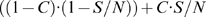, 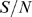 respectively. Pertinence scores are computed as follows: sensitivity score 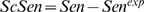, specificity score 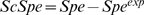, accuracy score 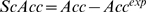 and PPV score 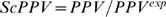.

To compare JET performance and the ET analysis described in [22], we used the Matthews' correlation coefficient (MCC) [23] defined as 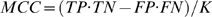 where 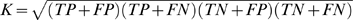.

When JET gives no answer (for example, due to an insufficient number of sequences retrieved by PSI-BLAST), then all interface residues are treated as negatives with 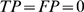, 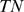 being the difference between the surface size and interface size (computed as the number of residues) and 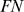 being the interface size. Notice that 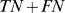 are all the negatives.

All evaluation scores reported in the Tables, Text S2, Text S3, and Text S5 are multiplied by 100.

### Dataset of Structures for Testing JET and Comparisons to ET

The Huang dataset of 62 protein complexes constituted of 43 homodimeric chains, 24 heterodimeric chains and 19 transient chains [4] has been used to test JET performance and to compare it to ET (see below). The PDB code, chain and size of all proteins in the Huang dataset are listed in Text S2. Some of the chains appear in complexes of different types: heterodimers and homodimers include four combinations of the same chains, and homodimers and transients include two combinations.

Several additional protein structures discussed in the text are listed in Text S3. All results reported in Tables, Text S1, Text S2, and Text S3 have been obtained with sequences retrieved from the PSI-BLAST server.

To check JET behavior on interfaces belonging to different functional categories, we used the Kanamori dataset of 265 interfaces which contains 72 signal transduction proteins, 43 enzymes, 19 inhibitors, 36 antibodies, 31 antigens, 64 other proteins [22]. This dataset was originally constituted to evaluate the possibility to employ information on residue conservation coming from ET to direct docking.

Structures of proteins and complexes used for the analysis were downloaded from Protein Data Bank http://www.rcsb.org/pdb/home/home.do.

### Comparison with ET

ET predictions (that is, residue average trace values and clusters) have been obtained using locally ET Viewer ( http://mammoth.bcm.tmc.edu/traceview/). ET default values are: 500 BLAST retrieved sequences, a sequence identity between 26–98% for retrieved sequences, a cut-off of 0.7 on the length of retrieved sequences, a maximum BLAST e-value at 0.05, a coverage of 25% for clustering residues belonging to the whole protein (not only those lying on the surface). Note that for small proteins, the 25% protein coverage corresponds essentially to surface coverage, but that, in general, one should expect ET to cover much less protein surface.

### Comparison with ET on Kanamori Dataset

ET predictions were taken from [22]. iJET was run with default values and complexes interfaces were evaluated with NACCESS. Six chains (1cdk:I, 1cdm:B, 1i4o:C, 1jdp:H, 1nrn:R and 1vrk:B) in Kanamori dataset were too small (≤20aa) to be evaluated with iJET and in this case the evaluation of the complex considered TP = 0 and FN = 0 for these chains.

### Implementation

JET has been implemented in Java and Java 3D. A list of all default values for JET parameters and instructions on how to use it is given in Text S4. The program can be found at http://www.ihes.fr/˜carbone/data.htm. JET output files can be visualized with available programs like VMD, used to generate all figures of protein structures in this article [24].

## Results

JET successfully addressed a series of problems inherent to the automatic prediction of protein interfaces and introduced for this a number of new conceptual features. We describe the novel contributions and conclude by validating JET on different types of interfaces.

### The Sequence Sampling Problem: A Solution by Cases

Large-scale predictions of interaction sites from evolutionary signals are highly sensitive to the degree of variability within the available sequences. The Huang dataset contains a pool of proteins which, overall, turns out to be quite well-sampled by a PSI-BLAST search. This resulted in an average of 358, 210, 61 and 29 sequences for the 20–39%, 40–59%, 60–79%, 80–98% identity classes for the whole set of proteins. There are however a few exceptions which are worth discussing since a large-scale approach needs to handle such cases appropriately. Notably, an adjustment of the number of trees and number of sequences in a tree is important to ensure the most appropriate sequence sampling within the trees.

#### Families of highly conserved proteins only: the case of 1n5y

A very high sequence identity between retrieved sequences implies too many residues will be characterized by a high trace value. A way to handle this situation is to retrieve more sequences until at least two identity classes are represented. No protein in the Huang dataset required retrieving more than 1000 sequences, see Text S2. There are however proteins such as DNA transferase 1n5y that only yield sequences in the class 80–98% among the first 1000 sequences retrieved by PSI-BLAST. This bias requires selecting a larger pool as demonstrated by the evaluation shown in Table 1. Very satisfactory results are achieved using iJET and increasing the pool size to 5000.

**Table 1:** JET, iJET, and ET evaluation on chain 1n5y:A.

Evaluation on Chain 1n5y:A									
Retr Seq	Tool	Sen	ScSen	PPV	ScPPV	Spe	ScSpe	Acc	ScAcc
1000	JET - cons	11.9	2.8	35.0	1.3	91.9	1.0	70.4	1.5
	JET - cons+pc	12.7	5.2	45.5	1.7	94.4	1.9	72.4	2.8
	iJET	7.6	2.4	39.1	1.5	95.6	0.9	72.0	1.3
5000	JET - cons	18.6	7.9	46.8	1.7	92.2	2.9	72.4	4.3
	JET - cons+pc	22.9	12.4	58.7	2.2	94.1	4.6	74.9	6.7
	iJET	**22.0**	**12.7**	**63.4**	**2.4**	**95.3**	**4.7**	**75.6**	**6.8**
478	ET	20.3	2.6	30.8	1.1	83.2	0.9	66.3	1.4

Sequence Identity of Retrieved Sequences for Chain 1n5y:A								
	5000 sequences				1000 sequences			
Sequence identity	20–39	40–59	60–79	80–98	20–39	40–59	60–79	80–98
# filtered sequences	0	0	44	2307	0	0	0	1000

Predictions for DNA transferase chain 1n5y:A when 1000 or 5000 sequences are retrieved using PSI-BLAST. Best predictions are obtained with iJET (in bold) run on 5000 retrieved sequences. JET results are improved when physical-chemical properties are taken into account (compare JET-cons with JET-cons+pc). For different classes of sequence identity we report the number of sequences collected in each class after filtering. The 478 sequences retrieved by ET with BLAST have sequence identities in the range 90–96%.

#### Families of mostly divergent proteins

Almost no sequences in the dataset we studied fall into this case. The B and C chains of cyclin dependent kinase 1g3n are exceptions that collect very few sequences with sequence identity >39%. Filtered sequences of chains 1g3n:B and 1g3n:C have an average sequence identity of 28.8 and 23.4, respectively. The transient interface is poorly detected for chain B, but reasonably well for chain C (with a 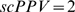). In such cases, performance is variable and depends strongly on the retrieved pool of sequences.

#### Very small families of related proteins

Some proteins might have very few retrieved sequences. In this case, JET will constructs a few small trees, namely, 10 trees of 10 sequences each. Under these extreme conditions, successful predictions seem to depend on a combination of two factors: good sequence variability (that is, the retrieved sequences should be neither too close nor too divergent) and a reasonable length (longer sequences should provide better results). Among the proteins analyzed here, the Shc PTB domain 1shc:A (195aa), the oncogene protein 1ycr:A (85aa), and the protein mimicry of DNA 1ugh:I (82aa) fall in this category. JET performs best on 1shc:A, the longest protein chain in this group, with all sequence identity classes represented. Physical-chemical properties (and not only conservation) play a role in the prediction (see Text S3). Similar observations hold for 1ycr:A. All retrieved sequences of 1ugh:I fall into class 20–39% and this suggests that JET's poor performance may be due to a combination of low sequence identity and insufficient sequence representation (see Text S2).

The retrieval of few sequences might induce JET to accept large e-values. For 1ugh:I, for instance, sequences of e-value 92 have been accepted. One might wonder about the biological meaning of such filtering choice, and 1ugh:I demonstrates that without such a lax condition, no prediction could be made. ET method failed to predict on this difficult example.

### The Interface Size Problem: A Parameterized Solution

Depending on the size of a protein we should expect that a different proportion of residues will belong to interfaces [20]. As discussed in Material and Methods, the clustering algorithm uses an estimation of the size of the expected interaction site as a function of the size of the protein. This estimation varies significantly for proteins of different sizes 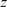. Roughly, 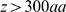 corresponds to an interface that covers <10% of the entire surface, 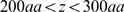 to <15%, 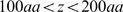 to <25% and 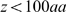 to a fraction varying (rapidly) from 25% to 90%. It might seem that for small proteins, JET covers a large proportion of the surface, but this has advantages as illustrated by the 85aa long Mdm2 protein chain 1ycr bound to the transactivation domain of p53. JET predictions cover 46% of the surface and by doing so, detect 71% of the interaction site, that is 10 residues interacting with P53 out of 16 (see comparisons with ET below).

### Better Predictions and Computational Advantage in Using Gibbs-like Sampling of Sequences

**Figure 6:**
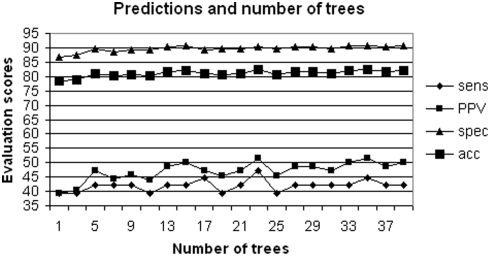
JET and number of distance trees. JET predictions (based on conservation and specific physical-chemical properties) for *Escherichia coli* aspartate transcarbamoylase structure 9atc:A have been evaluated on 625 sequences obtained with PSI-BLAST. Each of the 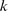 tree used contains 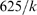 sequences. Each dot in the figure corresponds to a single run of JET.

One of the characteristics of JET is to use several distance trees of randomly sampled sequences instead of just one distance tree grouping all sequences recovered with PSI-BLAST. In Figure 6, we show the improvement in JET predictions solely due to dividing sequences amongst several trees. This is done by varying the number of trees 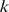, and by evaluating JET performance. (For each 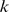, we ensure that JET treats roughly the same quantity of sequence information by requiring each tree to contain 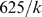 sequences. Note that due to a random choice of sequences for each tree, there is a high probability that the 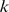 trees will share some common sequences). Improvements come from a consensus in residue trace values as a consequence of the degree of significance of a trace. This is determined by the number of trees used in the prediction. The plot shows better predictions for larger number of trees and also that the methodology leads to decreasing the noise due to incorrect alignments, the presence of non-homologous sequences in the pool, biased samples and so on.

**Figure 7:**
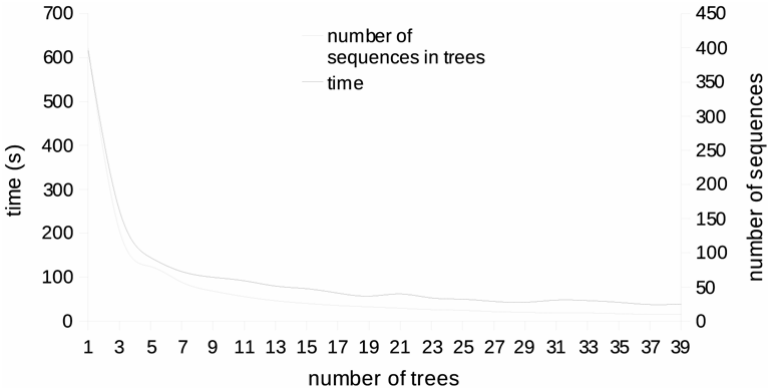
JET computational time. JET computational time (in seconds) has been evaluated for 400 sequences homologous with that of *Escherichia coli* aspartate transcarbamoylase 9atc:A. Each of the 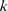 trees used contains 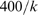 sequences, as indicated by the curve “number of sequences in trees”. For the evaluation, we used a Dual Intel Xeon (64-bit) 3.2GHz 2GB memory with Linux system.

Figure 7 illustrates the execution time of JET (excluding the PSI-BLAST step) when it is applied to the same pool of 400 sequences, but varying the number of trees. Sequences in the pool are all homologous to the sequence of chain 9atc:A. If 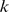 trees are considered, each tree contains 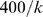 sequences which are randomly selected in the four identity classes as explained in Material and Methods. The plot shows that execution time is proportional to the number of sequences in the trees, with the major contribution coming from the CLUSTALW alignment.

### Looking at Surface Residues versus All Residues

**Figure 8:**
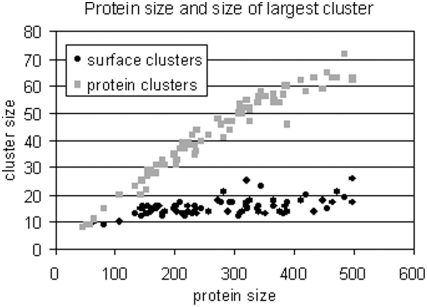
Protein size and size of the largest cluster. Sizes of the largest protein cluster (grey) and of the largest surface cluster (black) are plotted for all proteins in the Huang dataset. Size is defined by the number of residues.

Given a protein structure, JET estimates the size of the largest surface cluster for the protein (obtained by taking the largest cluster computed over 6000 iterations of the random clustering procedure). Based on the number of estimated residues, it predicts an interaction site of the appropriate size. The need for a structure-specific estimation results from the absence of a correlation between protein size and size of the largest surface cluster as illustrated in Figure 8 (black dots) for the Huang dataset. On the contrary, there is a linear correlation between protein size and cluster size when all protein residues are considered (see Figure 8, (grey dots), where random clustering is carried out on all protein residues and not only surface residues). Based on this property, [25] proposed a linear correlation and used it to predict the largest acceptable protein cluster for a given protein. Our analysis shows that cluster size predictions based on structure-specific estimations are better.

### Comparison between JET and ET: Improvement Due to the Clustering Procedure

JET is a prediction tool that uses evolutionary information to detect conserved interaction sites and was inspired by the Evolutionary Trace approach. Comparisons with ET are therefore necessary. The performance of JET and ET on the Huang dataset are presented in Text S2 for each protein and a synthesis is provided in Table 2 (compare lines “ET” and “JET-cons”) for homodimer, heterodimer and transient interfaces. The two systems perform in a comparable way when clustering is not applied. After clustering, JET covers 38% (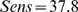) of the interface against 35% for ET. The interface residues predicted by JET correspond to real interface residues with a probability of 0.6 (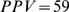) against 0.5 for ET. JET prediction scores are two times better than random predictions (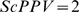). JET found 86% of residues which are not in the interface (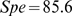) against the 84% for ET. The combination of these evaluating factors implies an average accuracy of 71% for JET against 68% for ET.

**Table 2:** JET, iJET, and ET evaluation on the Huang dataset.

	Sen	ScSen	PPV	ScPPV	Spe	ScSpe	Acc	ScAcc
**No Clustering**								
ET	35.4	14.0	50.4	1.6	83.9	5.1	68.4	7.8
JET – cons	34.7	13.3	50.3	1.6	84.5	5.8	67.6	7.5
**With Clustering**								
**Homodimers**								
ET	35.7	15.1	51.9	1.7	86.0	6.6	69.9	8.8
JET - cons	34.7	17.1	60.4	2.0	90.5	8.1	73.5	10.5
JET - cons+pc	37.9	18.9	61.2	2.1	89.5	8.4	73.1	11.0
iJET	**36.2**	**18.6**	**62.7**	**2.1**	**90.8**	**8.4**	**73.9**	**10.9**
**Heterodimers**								
ET	35.5	15.4	55.6	1.8	**86.3**	6.4	68.2	8.7
JET - cons	41.7	17.5	60.8	2.0	84.2	8.4	70.2	10.5
JET - cons+pc	47.9	21.1	59.6	1.9	83.8	10.5	71.2	12.6
iJET	**46.6**	**21.2**	**62.1**	**2.0**	**85.6**	**11.0**	**72.7**	**13.0**
**Transients**								
ET	29.7	10.5	50.7	1.5	**82.2**	1.4	63.8	5.7
JET - cons	38.9	12.9	57.5	1.8	77.8	3.9	67.0	6.6
JET - cons+pc	39.7	12.9	55.9	1.7	78.3	5.1	67.3	7.0
iJET	**37.5**	**14.0**	**59.1**	**1.9**	81.7	**5.1**	**68.2**	**7.6**
**All Confounded**								
ET	34.2	14.2	51.8	1.7	85.1	5.2	68.5	7.9
JET – cons	37.9	16.4	59.0	2.0	85.6	7.1	71.4	9.5
JET - cons+pc	41.6	18.4	58.4	1.9	84.9	8.1	71.6	10.5
iJET	**39.8**	**18.5**	**60.5**	**2.0**	**86.9**	**8.2**	**72.6**	**10.6**

Comparison of JET and ET without clustering (top table) and with clustering (bottom table). Without clustering, JET and ET performance is comparable. JET performance with clustering is computed when signals of conservation alone (JET - cons) and mixed with information on physical-chemical (JET - cons+pc) properties of residues are considered. Results in the Table are averages of single runs of JET on proteins in the Huang dataset. Performance of iJET is also presented for clustered residues which have been obtained by a consensus of 7 runs over 10. Average performance is computed on homodimer, heterodimer and transient proteins in the Huang dataset. Average values computed for all proteins (all confounded) are given; proteins belonging to different categories (due to multiple chains establishing both homodimer and heterodimer interfaces for instance), are only counted once. For each type of interface, ET and iJET are compared and bold characters indicate best performance.

Differences in ET and JET performance with and without clustering suggests that the clustering procedure employed in ET is less successful than that proposed here.

In [10], it is argued that ET works best for families of homologous proteins with sequence identities higher than 40%. JET correctly detects functional sites of protein families well below this threshold. In Text S2, we provide the number of sequences retrieved with PSI-BLAST and the sequence identity classes for all proteins in the Huang dataset. For a large majority of these proteins, most retrieved sequences fall into the 20–39% class.

**Figure 9:**
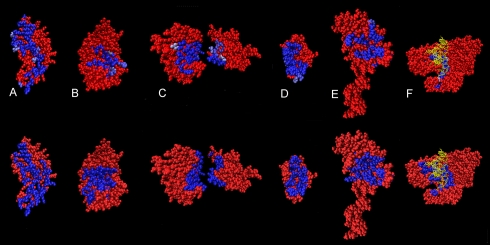
iJET predictions on several types of interfaces. Structures: allophycocyanin 1all:B (A), phosphotransferase 1apm:E (B), human CDC42 gene regulation protein 1grn:AB (C), oncogene protein 1ycr (D), signal transduction protein 1shc (E), large fragment of *Thermus aquaticus* DNA polymerase I 2ktq (with the DNA chain in yellow) (F). Top: iJET predictions with residues occurring at least 7 times out of 10 runs highlighted using a blue scale. Dark blue corresponds to 10 runs (the majority of residues in the figure). Bottom: experimental interaction sites.

For small proteins, the usage of an adapted curve (discussed above, see Figure 4) for evaluating protein coverage, also improves JET performance with respect to ET. An example is the Mdm2 protein chain 1ycr:A (discussed above, see also Figure 9D) where a 46% JET coverage contrasts with a 24.6% ET coverage, and results in the detection of 71% of the interaction sites (10 residues out of 16 interacting with P53) against only 41% for ET (5 residues out of 16). To understand this contrast, it is important to look at the scores 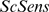, 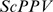, 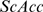 and 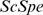 listed in Text S3, which describe behavior of the two approaches compared to a random choice of residues. Note that in the case of 1ycr:A, most of the 25% residues covered by ET are surface residues, since the chain is small.

### Comparison between JET and ET: Improvement Due to the Integration of Physical-Chemical Properties

**Figure 10:**
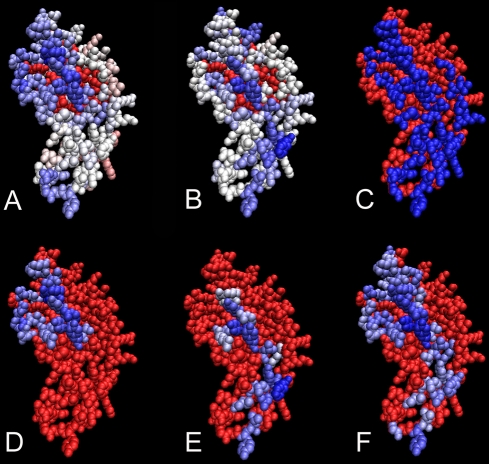
JET prediction of an interaction site based on the combination of conservation and physical-chemical signals. Allophycocyanin structure (PDB file 1all:B). Top: residues are colored from blue (strong signal) to red (low signal) passing through white by conservation (A) and by physical-chemical properties (B), with inaccessible residues in red. The experimental interaction site is blue in (C). Bottom: cluster seeds computed based on conservation (D) and based on physical-chemical properties (E) are colored from blue to white accordingly to 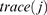 and 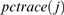 values respectively. Prediction (F) is computed by extending both seeds in (D) and (E); colors map mixed trace values.

Over the Huang dataset, a considerable improvement of JET performance is shown in Table 2 (compare lines “ET” and “JET - cons+pc”) when clustering is carried out on mixed traces, coupling both conservation signals and physical-chemical properties (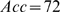, 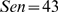, 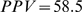 and 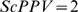). In this way JET predictions improve considerably, as seen in the allophycocyanin structure 1all:B (Figure 10 and Text S3) where by using physical-chemical properties together with conservation, JET detects 66.7% of the interaction site, while conservation alone only detects 51.1%. ET detects 40% of the site.

**Figure 11:**
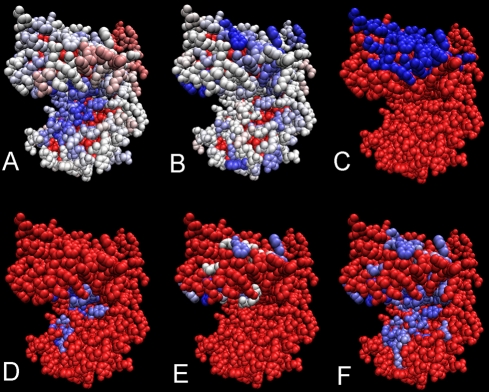
JET prediction of several interaction sites based on the combination of conservation and physical-chemical signals. Leucine dehydrogenase structure from *Bacillus sphaericus* (PDB file 1leh). (A–E) are as in Figure 10. JET predicts two sites (F), the protein interaction site (on the top) and the ligand binding site.

The leucine dehydrogenase structure 1leh in Figure 11, again illustrates that using physical-chemical properties improves the detection of interaction sites: the ligand site is constituted by very conserved residues, while the protein interface displays strong physical-chemical signals. The latter, combined with residue conservation, help JET to extract a suitable cluster describing the interaction site. ET fails to detect the site (see Text S3). Here, the PDB file used did not contain information on the ligand interaction and thus residues predicted to belong to the ligand interface were erroneously classed as false positives by our automatic procedure. This example illustrates the difficulty of a large-scale evaluation of a prediction system.

### Variability Due to Gibbs-like Sampling and Evaluation of Residues by Consensus: iJET

**Figure 12:**
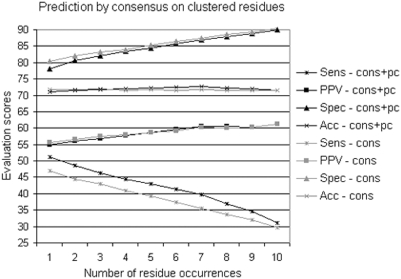
iJET consensus on clustered residues. iJET predictions for the Huang dataset using conservation (grey), and conservation and physical-chemical properties (black). Evaluations concern residues occurring 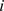 times over 10 runs of JET. For each 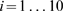, we plot the average evaluation over all proteins in the Huang datset.

To check whether clusters predicted in different runs of JET represent a consensus or not we iterated JET 10 times and analyzed its performance. Namely, given a protein, we considered a *consensus prediction* defined as the ensemble of residues that appear in a cluster at least 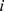 times, for 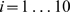, out of the 10 iterations of JET on the protein structure. We then evaluated JET on each protein of the Huang dataset for increasing values of 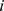 (see Figure 12). As expected, for increasing 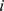, predictions show a better PPV, but a worse sensitivity. This corresponds to an increased selectivity in choosing residues to belong to clusters. If conservation is coupled with physical-chemical properties, then specificity, accuracy and PPV curves show the best prediction at 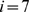. The evaluation of JET iterated 7 times on the Huang dataset is presented in Table 2 (line “iJET”).

The take-home message from this study is that different runs of JET are likely to provide slightly different outcomes and that a robust prediction of residues at the interface can be drawn from 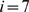 iterations. In this case, JET obtains very good average scores: 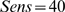, 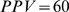, 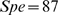, 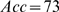. Compare it with ET: 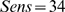, 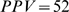, 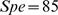, 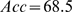. JET is consistently better for homodimers, heterodimers and transients interfaces. It is important to stress that the iterative procedure suggests a list of residues that do not necessarily form clusters (as defined above), but patches of residues (and possibly isolated residues) that have been consistently (that is, in most JET runs) been classed as being part of an interaction interface. The iterated version of JET (based on 10 iterations) is called iJET.

**Figure 13:**
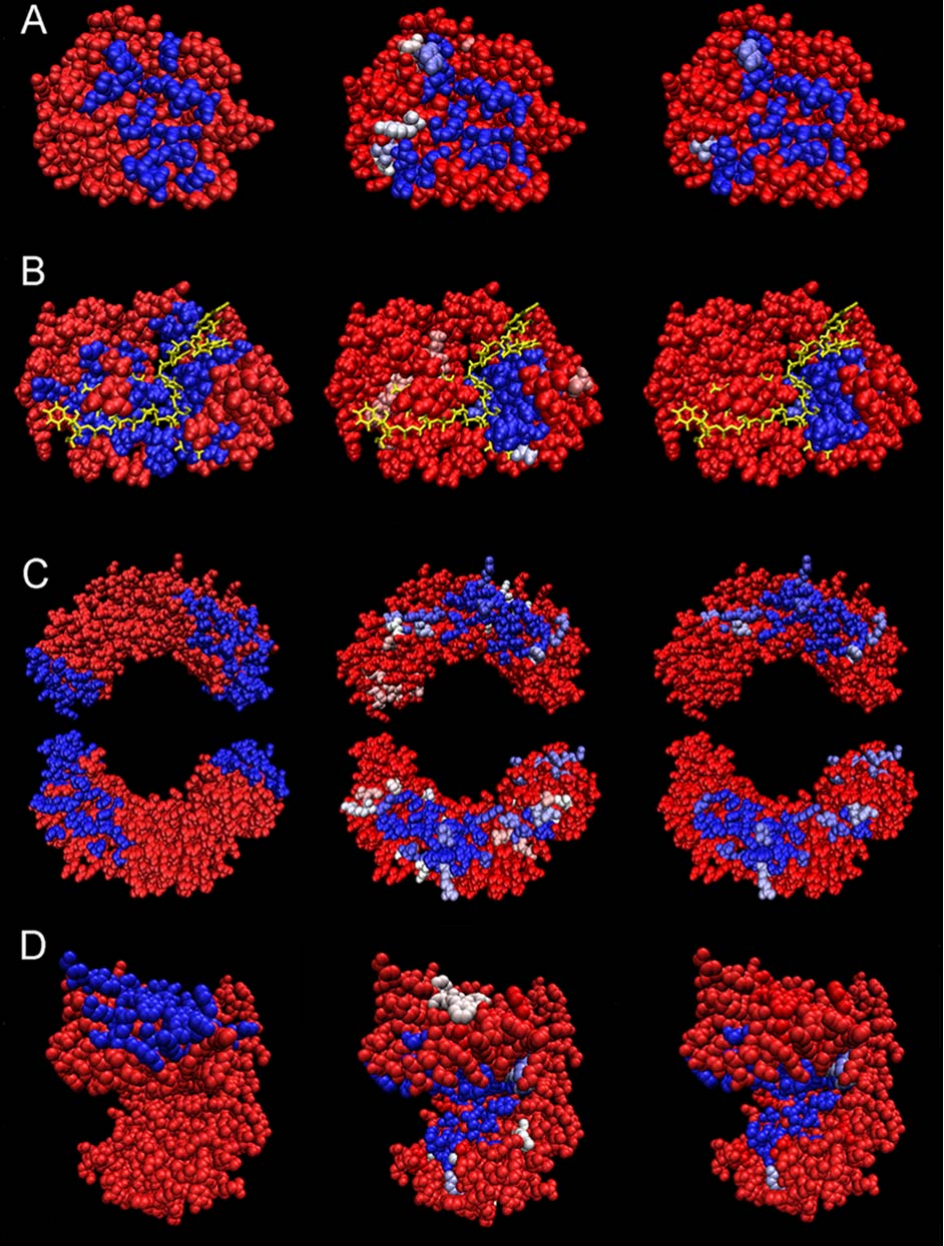
iJET predictions and consensus on residues. Structures: *β*-trypsin proteinase 2ptc (A), RNA-binding protein 2cjk (with the RNA chain in yellow) (B), nucleotidyltransferase 2pol (C), oxidoreductase 1leh (D). Left: experimental interaction site (blue). Center: residues appeared at least 2, 2, 3, and 4 times respectively for structures (A–D) over 10 iterations of JET. Right: residues appeared 7/10 times (that is, at least 7 times out of 10 iterations of JET). Central and right columns: predicted residues are colored from blue to pink depending on number of iterations selecting the residue out of 10 runs; dark blue for 10/10, white for 7/10 and dark pink for 2/10.

In Figure 13, we illustrate iJET behavior for different values of 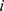 on several protein structures. Structures B, C, D show that for 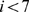 (column in the middle) we could detect residues belonging to the real interface that are missed for 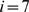 (right hand column). This means that in a single protein analysis, it could be worthwhile for the user to try different values of 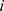 and evaluate the best 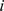 ad hoc. For instance, for the structures of Figure 13, residues appearing <7 times (colored in pink) are not always interface residues (see A and B). For a large-scale analysis such tests are impossible and the value of 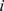 needs be fixed. As we have shown, setting 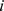 at 7 is appropriate for the Huang dataset.

In Text S2 and Text S3, a comparison between iJET and single-run JET (using combined conservation signals and physical-chemical residue properties) shows that single-run JET can produce better results than the average obtained with iJET. The reason for this lies in the variable information content of pools of sequences retrieved by PSI-BLAST. This suggests that many of the sequences may be noisy in relation to the interaction site, although this noise can be eliminated in certain runs. Note that if JET is run on a single tree constructed out of sequences retrieved by PSI-BLAST, the result remains identical for all iterations. Computational strategies to ameliorate iJET will be discussed elsewhere.

### The Protein Length Effect

Small proteins are clearly more difficult to analyze than large ones. This is shown in Table 3 that revisits the performance of iJET presented in Table 2 with respect to protein length. Small proteins (with <200aa) display a less stable behavior compared to larger ones (≥200aa): evaluation scores for the two classes of large proteins in Table 2 are closer than for the two classes of small proteins. Specificity and accuracy remain essentially unchanged for large proteins and much lower values are attaint for small proteins. As expected, best sensibility and PPV are reached for small proteins due to a large coverage (see Figure 4).

**Table 3:** iJET performance by protein length.

	Sens	PPV	Spe	Acc
1–99	**58.39**	**75.42**	58.39	63.73
100–199	46.24	61.32	81.81	66.95
200–299	41.34	66.47	**91.56**	73.26
> = 300	32.12	54.17	**91.31**	**77.07**

Evaluation of iJET on all proteins in the Huang dataset, organized by amino-acid length. Four classes are considered. Highest scores (by columns) are in bold.

### Comparison with Consurf and Rate4Site

iJET has been compared to Consurf [26] and Rate4Site [27] on the Src SH2 domain of the 1fmk structure discussed in [26],[27]. iJET run 10 times on sequences which were automatically downloaded from the PSI-BLAST site, and where each residue trace value is the maximum trace value over the 10 runs.

**Figure 14:**
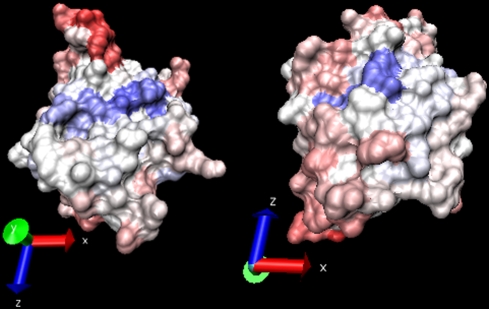
iJET prediction of interaction sites for the SH2 domain of the human tyrosine kinase C-SRC. Structure: 1fmk. Residues are colored from blue to red passing through white by conservation and physical-chemical properties using maximal trace values over 10 runs. Left: SH2 and C-tail of tyrosine kinase domain interaction site (blue region). Right: TRP148 (blue) highlights the SH2–SH3 domain site.

**Figure 15:**
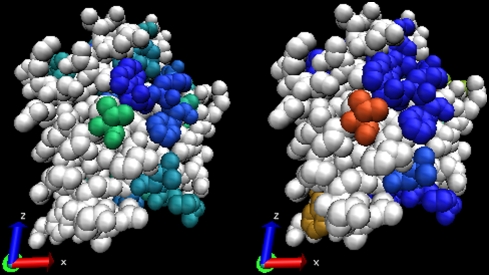
JET and iJET predictions of interaction sites calculated on close SH2 homologues. Structure: 1fmk. Non selected residues are colored white. Colors are set on a scale from red to blue passing through green. Left: cluster predicted by JET covering the three known interacting sites of the human SRC SH2 domain; colors represent the mixed trace value of a residue. Right: iJET predictions over 10 runs. Colors represent the number of runs selecting a residue. Notice the residue located in the middle of the protein face: it belongs to the linker loop interface, it displays average conservation (green, left), and it has been detected once over 10 runs (red, right).

Consurf and Rate4Site run on 233 homologous sequences (Figures 2, 3A, and 3B in [27]). The site between SH2 and the C-tail of the tyrosine kinase domain predicted by iJET is comparable to Consurf and Rate4Site predictions (compare Figures 2 and 3 in [27] and Figure 14, left). The three systems do not detect any residue in the SH2-kinase domain interface nor in the SH2-linker loop site. iJET detects as important (due to both conservation and physical-chemical properties) residue TRP148 sitting in the SH2–SH3 domain interface (Figure 14, right). Consurf detects no conserved residue, while Rate4Site identifies the site. By using 34 close SH2 homologues from the Src family [27], clear signals of conservation belonging to the multiple interaction sites are detected by the three systems. This is expected since the Src family is highly conserved. In this case, SH2–SH3 domain and SH2-kinase domain are well detected (see Figure 3 in [27] and Figure 15). The SH2-linker loop interface is detected as highly variable by Consurf while Rate4Site assigns to it an average conservation. iJET correctly detects the site even though it assigns to it a signal of average strength (see Figure 15, left). This is possible because of the cluster seed extension procedure in the clustering algorithm that does not require a residue to be conserved to belong to a cluster. It is important to see that no other residue located close to the conserved region is erroneously detected by iJET (see Figure 15, right).

In conclusion, JET appears to perform better than Consurf and slightly less well than Rate4Site for the SH2–SH3 site. It demonstrate to be a successful platform for detecting very difficult signals like the linker loop interaction, where both Consurf and Rate4Site failed. Compared to Consurf, we can observe that it is able to detect important residues (such as TRP148) starting from a very mixed pool of sequences. It is interesting to notice that iJET and Rate4Site agree on the very variable residues, contrary to Consurf prediction of variability (see residues on top of the structure in Figure 14, left, and on bottom of the structure in Figure 14, right, and compare them to Figures 2 and 3 in [27]).

### Comparison with siteFiNDER|3D, Consurf, and ET Viewer 2.0

**Figure 16:**
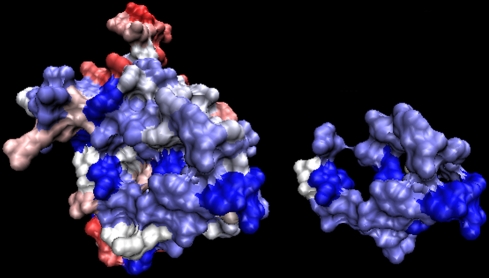
N-terminal domain of MukB. Structure: 1qhl:A. Residues are colored as in Figure 14. Left: full structure. Right: all residues predicted by iJET and selected on at least 8 JET runs; the Walker-A motif of the putative G-loop is detected.

iJET is compared to siteFiNDER|3D [28], Consurf and ET Viewer 2.0 on the N-terminal domain of MukB (1qhl:A). iJET run on its own set of homologous proteins selected from its PSI-BLAST output, and important residues are defined to appear at least 8 times over the 10 JET runs. iJET pool of sequences gave rise to the expected prediction with 29 residues out of 227 residues in the chain, exhibiting a higher specificity than siteFiNDER|3D evaluated on its own dataset of sequences (45 over 227). The important residues determined by iJET are all clustered around the putative G-loop and include Gly34, Asn36, Gly37 and Lys40 from the Walker-A motif (Figure 16). This result shows the high specificity of iJET. Consurf run on its own set of sequences detects the Walker-A site but with a specificity of 37 out of 227 residues, therefore lower than iJET. ET Viewer 2.0 run on its own dataset failed to make a useful prediction. As Consurf and ET Viewer 2.0 (when these latter are applied to some well chosen dataset of sequences), JET detects as conserved other residues which lie in the same face of the molecule (like Glu202 and Tyr206), and this suggests a possible role in dimerization of MukB.

### Prediction of Functionally Specific Residues and Comparison with SCORECONS

**Figure 17:**
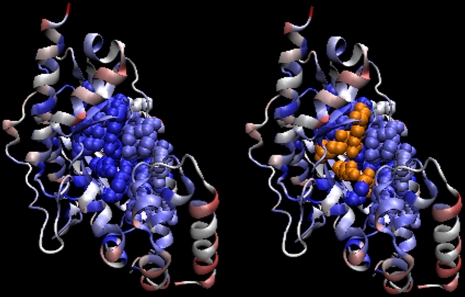
Arginine kinase. Structure: 1bg0. Residues are colored as in Figure 14. Left: important residues predicted by iJET are plotted in space-fill view. Right: functionally important residues (orange).

We analyzed the structure of Arginine kinase ( 1bg0) discussed in [29]. We run iJET and we selected as important those residues that appear in JET clusters for 10 runs (Figure 17). Notice that this is a very restricting condition for selection. iJET detected as important (and conserved) and as belonging to the interaction site the functionally specific residues GLU225, ARG229, ARG280 and ARG309 [30] (it misses ARG126). These residues, as well as all others forming the interaction pocket, are not detected as conserved in [29], using SCORECONS [31] and the program alignment MUSCLE as input. Also, [29] detects only 2 over 5 residues as functionally important. This example confirms the results obtained at large scale on the Huang dataset, where we see that binding pockets are usually well detected. It shows JET accuracy in detecting conservation signals.

### JET Detection of Very Different Interaction Sites

JET is capable of detecting very different types of interface, as illustrated here with several case studies. Some belong to the Huang dataset (Text S2) and others are listed in Text S3. See Figures 13 and 9. Comparison with ET provides an evaluation of the power of JET in these cases. A large-scale analysis of interfaces with different functional classification is realized on the Kanamori dataset (Text S5) and follows.

#### Heterodimers, homodimers, and transients

Proteins in the Huang dataset are organized in heterodimer, homodimer and transient interfaces. We observe a similar behavior of JET (and iJET) on heterodimers and homodimers (Table 2). As expected, transient interfaces are more difficult to predict [6] as shown by the lower iJET evaluation scores obtained for transients compared to heterodimers and homodimers. The same observation holds for ET.

#### Ligand sites and protein interfaces

Ligand interaction sites are often pockets involving very conserved residues. In contrast, protein interface sites are less conserved regions and their residues are often characterized by specific physical-chemical properties, especially hydrophobicity. This point is nicely illustrated by the leucine dehydrogenase structure 1leh in Figure 11, which undergoes a conformational change when leucine and NADH bind to the ligand pocket. The well-conserved pocket is shown in (A) and the protein interaction site is mostly characterized by specific physical-chemical properties as shown in (B). The conserved ligand site is very large and the strong conservation signal prevents JET from detecting the much weaker signal associated with the protein interface. The coupling of conserved residues and residues displaying specific physical-chemical properties is thus crucial for detection of the interface in 1leh (Text S3).

Two other examples involve the GTP-binding chain 1grn:A (Figure 9 C) and the D-amino acid aminotransferase 1daa which also contain a ligand-binding site and an interaction site. In these two cases, protein and ligand sites overlap to some extent. Thus, the ligand site of 1daa lies in a pocket partially included in the interaction site with the protein partner. Residues 50, 145, 204, 205 and 241 form the ligand interaction site and they are all correctly predicted. The interaction site is also successfully detected (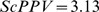) as described in Text S2. In 1grn, the ligand and protein interaction sites are located side-by-side. JET correctly predicts residues 13, 15, 16, 17, 18, 159 and 160 that form the ligand site and only misses residue 118. The protein interface is also well detected (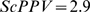). See Text S3.

#### Multiple protein-protein binding sites in the same protein

The nucleotidyltransferase 2pol is characterized by four distinct interfaces (see Figure 5), two interacting with its homodimeric partner and two with chains 1jql:B and 1unn:CD. The conservation signal is very strong for the 1jql:B interface and for one of the homodimeric interfaces. They are both found by iJET with a consensus of 7 runs over 10 (see Figure 13C, right column). The interface with 1unn:CD is partially found with 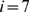 and improved with 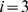.

For multiple sites, the fraction of interface residues compared to the surface size might be larger than the one estimated by the curve in Figure 4, used by our clustering method. This amounts to an under-estimation of the coverage threshold used in the algorithm and to a loss of weak conservation signals (see Text S2). This happens for one of the homodimeric interfaces that is not found even though its conservation is visible in Figure 5B.

#### Receptor/inhibitor pockets

Three receptor/inhibitor complexes have been analyzed: 1ugh, 2ptc and 1k9o. For all three, the receptor site forms a conserved pocket and is very well predicted by JET, while the inhibitor interface is not. The three proteins display catalytic activity within the conserved pockets and this is consistent with the presence of the strong signals of conservation that we generally observe for ligand binding sites. The inhibitors 1ugh:I (82aa) and 2ptc:I (58aa) are short peptide chains while 1k9o:I (376aa) is a long chain. This suggests that a small length should not be considered as the reason for the failure. Multiple interactions of the inhibitor with several proteins might rather explain the lack of strong conservation, while the discrimination of interacting partners might rely more on the geometrical shape of the inhibitor.

Results are given in Text S2 and Text S3. (Scores of 2ptc:I: 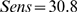, 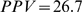, 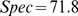, 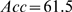)

#### Proteins binding to DNA and RNA

As for ligand interactions, interaction sites of a protein with DNA or RNA appear to be rather conserved as illustrated in the structures of the fragment of DNA polymerase I 2ktq (Figure 9F) and of an RNA-binding protein 2cjk (Figure 13B). Most conserved residues are those interacting with nucleic acids.

For 2cjk, there are three regions that enter in contact with RNA for the recognition of the specific termination signal AUAUAU. Two of them lie on the conserved site detected by iJET with 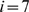 (see Figure 13B, right column) and the third one (colored pink) is detected with 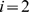 (middle column).

For 2ktq, the strong conservation signal corresponds to a ligand site (the five residues interacting with the ligand are all found) and to roughly half of the residues interacting with DNA. In contrast, the nucleotidyltransferase 2pol does not show any conservation of the residues in contact with the DNA. In fact, for this protein there is no need for specific recognition, its function depending on residue charges which bind DNA, but allow it to slide (see Figure 5 and Figure 13C).

### Large-Scale Comparison of iJET Behavior on Different Functional Classes of Interfaces

**Figure 18:**
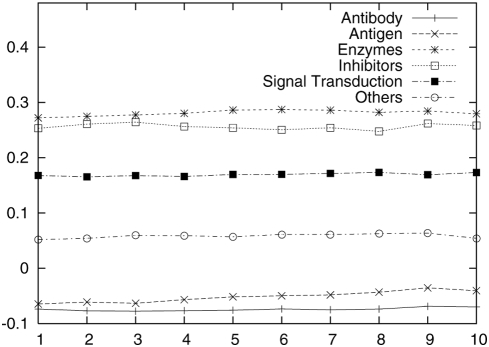
Matthews' Correlation Coefficients of iJET on Kanamori dataset. iJET evaluation based on MCC (y-axis) on different functional classes of proteins of Kanamori dataset, with 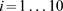 (x-axis).

Even though JET detects several interaction sites and any evaluation is difficult, we compared it with the performance of ET on the Kanamori dataset of proteins organized in functional classes, where specific pairwise interactions were targeted. The overall evaluation scores attaint by iJET cannot be very good due a potentially erroneous increase of false positives coming from JET detection of multiple interaction sites, but an honest comparison of iJET to ET can be drawn on functional classes following [22]. Namely, we considered 265 protein interfaces belonging to different functional classes: signal transduction proteins, enzymes, inhibitors, antibodies, antigens and others [22], and considered as positives, the residues in the two interacting chains that belong to the interface. We found that iJET performs well in signal transduction proteins, enzymes and inhibitors, while a poor behavior is recorded on antigen and antibody interface predictions (see Table 4, Figure 18, and Text S5). We observe an improvement with iJET compared to ET [22]. The striking difference between our analysis and [22] is that for us inhibitors work essentially as well as enzymes. The MCC computed by [22] on the inhibitors class is −0.01 (with a standard deviation of 0.14) while we obtain a MCC of 0.26 (with a standard deviation of 0.11) which is comparable to the MCC of 0.28 (and standard deviation of 0.13) obtained for enzymes. Similar prediction quality for enzymes and inhibitors is not explainable by similar evolutionary pressure of enzyme-inhibitor partners since the two protein classes display asymmetric residue conservation [22],[32]. (See remarks above on receptor/inhibitor pockets.) iJET good performance on the inhibitors class might be due to the fact that iJET takes into account also physical-chemical properties for residue evaluation, and that it detects interaction sites accordingly to the biological hypothesis that clusters are formed by a conserved internal core surrounded by successively less conserved layers of residues. A careful analysis of the distribution of conserved residues on the inhibitor interaction sites should be able to clear out this point, but this will be done somewhere else. In conclusion, our finding support the crossed usage of iJET predictions with docking algorithms, leading to a reduction of the docking search space for signal transduction proteins, enzymes and inhibitors.

**Table 4:** iJET and ET evaluation on the Kanamori dataset.

Category	iJET		ET Kanamori	
	MCC	SD	MCC	SD
Enzymes	0.28	0.13	0.24	0.14
Inhibitors	0.26	0.11	−0.01	0.14
Signal transduction	0.17	0.17	0.14	0.22
Antigen	−0.04	0.12	0.02	0.13
Antibody	−0.07	0.08	−0.05	0.09
Others	0.06	0.16	0.02	0.19

The Matthews correlation coefficients (MCC) computed for iJET (with i = 9) on all protein complexes of the Kanamori dataset (with no redundancies). The standard deviation (SD) of the distribution is also indicated. MCC and SD computed on ET in [22] are reported for comparison.

## Discussion

### Conserved Patches

Conserved patches of residues on a protein surface can help to suggest the location of an interaction site. We have tested the evolutionary hypothesis that interaction sites are constituted by a conserved internal core, surrounded by successively less conserved layers of residues. Based on this hypothesis we were able to develop a new criterion for extending conserved patches (that is “cluster seeds”), improving predictions of realistic interface clusters. The impact of this extension step in the algorithm is nicely illustrated in Figure 15 where a residue belonging to the linker loop interface of SH2 is detected by the extension and remains non predicted by other systems.

By making multiple iterations, iJET predicts 40% of the interfaces for proteins within the Huang dataset (with 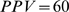, 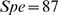 and 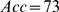), and more than 50% of the interfaces for proteins in Text S3 (with 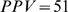, 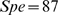 and 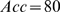). For more than a quarter of the proteins in Huang dataset, more than 50% of their sites are correctly predicted, and for 6 out of the 12 proteins in Text S3, 60% of the true site is identified by iJET.

### Physical-Chemical Composition of Protein-Protein Interaction Sites

We tested the evolutionary hypothesis that specific physical-chemical properties of residues forming interaction sites should co-exist with signals of residue conservation. We were able to show that a combination of conservation signals (even if low) and physico-chemical interface propensity values indeed leads to successful predictions. Future developments of JET will include an intelligent detection of patches satisfying specific physical-chemical properties based on propensity values differentiating multiple types of interaction [6].

### Appropriate Hits for Different Questions

JET and iJET can be used for large-scale analysis or as platforms to make *in silico* experiments on protein interfaces. These latter are possible due to the flexible parameterization provided by the system. Each step of JET can be monitored and improved by an accurate ad hoc understanding of the protein under study (this might end up into an explicit consideration of protein length, availability of homologous sequences, distribution of homologous sequences in sequence identity classes, expected conservation, etc.). The first hand information coming from a run of JET are the clusters that it provides. Notice that for large-scale comparison of JET and ET, we considered a hit to be the set of clusters issued by a single run of JET. For comparison with iJET, we considered a hit to be the set of clustered residues issued by iJET, with 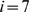 (pertinency of 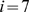 is discussed above). In single protein analysis, we might want to look for functionally specific residues, and it might be more appropriate to adopt very selective conditions, for instance by asking for a residue to appear in 10/10 clusters. If the aim is to discriminate between residue importance, it might be useful to use the maximal mixed trace for residues issued over 10 runs of JET, or as before, to select as important those residues appearing in 10/10 clusters. These measures are easily accessible to the user in the output files. Examples of the application of these criteria to single proteins are discussed in Results.

### Difficulties in the Evaluation of JET

Multiple interaction sites often occur on a protein surface and this makes evaluating JET difficult since only some of these sites may be experimentally characterized. JET is nevertheless capable of detecting all residues patches which are susceptible to be involved in interactions with other ligands or macromolecules. An example illustrating this point is leucine dehydrogenase 1leh (see Figure 11) Which has both a protein-protein interface and a ligand-binding pocket. The absence of information on the conserved pocket in the corresponding PDB file leads to apparent false positives when JET is used automatically (see Text S3), but such information can be valuable and can be used by the biologist in formulating new hypotheses.

### Applying JET

Lastly, it is remarked that JET can be applied to protein sequences for which the structure is unknown, if the structure of a homologous protein is available. This approach can again be valuable to the biologist, notably in guiding site-specific mutagenesis experiments [33].

## Supporting Information

Text S1 Propensity values
(0.05 MB PDF)

Text S2 ET, JET and iJET performance on the Huang dataset
(0.16 MB PDF)

Text S3 JET and iJET performance on a pool of selected proteins discussed in the article
(0.08 MB PDF)

Text S4 JET program information
(0.09 MB PDF)

Text S5 iJET performance on the Kanamori dataset
(0.10 MB PDF)
