# Supplementary material for: Joint Evolutionary Trees: A Large-Scale Method To Predict Protein Interfaces Based on Sequence Sampling
Source: PLoS Comput Biol. 2009 Jan 23;5(1):e1000267. doi: 10.1371/journal.pcbi.1000267 (PMC2613531; doi:10.1371/journal.pcbi.1000267)
Supplement: Text S1 — Propensity values (0.05 MB PDF) [file pcbi.1000267.s001.pdf]

Propensity values

Propensity Values									
W	I	F	L	C	M	V	Y	P	A
2.19	1.41	2.21	1.56	1.42	1.46	1.25	1.76	1.03	0.85
T	H	G	S	Q	N	E	D	K	R
0.7	1.3	0.9	0.8	1.0	0.9	0.7	0.7	0.6	1.1

TAB. 1 – Propensity values describing physical-chemical properties of residues at the interface as estimated in (Nagi and Braun 2007). A value ≥ 1 suggests that a residue most likely belongs to an interface rather than outside of it.
