# Supplementary material for: Joint Evolutionary Trees: A Large-Scale Method To Predict Protein Interfaces Based on Sequence Sampling
Source: PLoS Comput Biol. 2009 Jan 23;5(1):e1000267. doi: 10.1371/journal.pcbi.1000267 (PMC2613531; doi:10.1371/journal.pcbi.1000267)
Supplement: Text S2 — ET, JET and iJET performance on the Huang dataset (0.16 MB PDF) [file pcbi.1000267.s002.pdf]

ET, JET and iJET performance on the Huang dataset

Homodimers																
	JET trace results								ET trace results							
pdbCode	Sen	ScSen	PPV	ScPPV	Spe	ScSpe	Acc	ScAcc	Sen	ScSen	PPV	ScPPV	Spe	ScSpe	Acc	ScAcc
1bnc :A	30.6	9.5	33.8	1.45	81.8	2.9	69.8	4.4	33.3	10.9	34.8	1.49	80.9	3.3	69.8	5.1
1daa :A	48.5	26.8	69.6	2.23	90.4	12.1	77.4	16.7	53.0	30.9	74.5	2.39	91.8	13.9	79.7	19.2
1dpg :A	41.7	19.4	43.8	1.87	83.6	5.9	73.8	9.1	41.7	20.2	45.5	1.94	84.7	6.2	74.7	9.5
1ecp :B	36.6	13.4	76.9	1.58	89.5	12.7	63.7	13.1	39.0	16.4	84.2	1.73	93.0	15.6	66.7	16.0
1efu :A	31.2	7.3	37.5	1.31	79.1	2.9	65.3	4.2	37.7	11.9	42.0	1.46	79.1	4.8	67.2	6.8
1efu :B	45.2	21.9	60.0	1.94	86.5	9.8	73.7	13.5	53.4	29.7	69.6	2.25	89.6	13.3	78.4	18.4
1frp :A	35.3	14.3	63.8	1.68	87.8	8.8	67.9	10.9	38.8	18.7	73.3	1.93	91.4	11.5	71.4	14.2
1fuq :A	61.1	36.9	41.3	2.53	83.0	7.2	79.5	12.1	66.7	41.6	43.4	2.66	83.0	8.1	80.4	13.6
1gdh :A	29.0	9.1	41.7	1.46	83.7	3.6	68.0	5.2	26.1	5.8	36.7	1.28	82.0	2.3	66.0	3.3
1ges :A	46.1	22.4	65.4	1.95	87.7	11.3	73.7	15.1	47.8	23.3	65.5	1.95	87.2	11.8	74.0	15.6
1glq :A	34.9	14.4	45.5	1.7	84.7	5.2	71.4	7.7	37.2	14.2	43.2	1.62	82.2	5.2	70.2	7.6
1gpm :B	30.2	6.1	29.2	1.25	77.7	1.9	66.7	2.8	32.6	8.2	31.1	1.33	78.1	2.5	67.5	3.8
1hur :A	43.2	22.0	67.9	2.04	89.8	11.0	74.2	14.6	43.2	22.7	70.4	2.11	90.9	11.4	75.0	15.2
1hyh :A	27.4	4.0	57.1	1.17	80.5	3.8	54.6	3.9	29.1	4.5	57.6	1.18	79.7	4.3	55.0	4.4
1ids :A	30.3	10.2	62.5	1.51	87.1	7.2	63.5	8.4	22.7	2.6	46.9	1.13	81.7	1.8	57.2	2.1
1ies :B	21.1	2.3	64.0	1.12	84.2	3.0	48.1	2.6	22.4	4.3	70.8	1.24	87.7	5.8	50.4	4.9
1leh :A	3.7	-16.2	1.9	0.19	78.3	-1.8	70.8	-3.2	7.4	-12.2	3.8	0.38	79.1	-1.3	72.0	-2.4
1mas :A	34.6	19.5	58.1	2.29	91.5	6.6	77.1	9.9	28.8	11.3	41.7	1.64	86.3	3.8	71.7	5.7
1mld :A	49.2	25.5	54.7	2.08	85.5	9.1	75.9	13.4	54.2	30.1	59.3	2.25	86.7	10.8	78.1	15.9
1nhk :L	33.3	9.7	46.2	1.41	81.1	4.7	65.5	6.3	36.1	12.5	50.0	1.53	82.4	6.1	67.3	8.2
1nqv :A	29.9	6.4	74.1	1.27	85.4	8.9	53.0	7.4	31.3	7.0	75.0	1.29	85.4	9.8	53.9	8.2
1oro :A	48.9	22.3	51.2	1.84	82.1	8.6	72.8	12.4	53.3	27.4	57.1	2.06	84.6	10.5	75.9	15.2
1osj :A	42.4	19.6	49.1	1.86	84.2	7.0	73.2	10.4	45.5	22.7	52.6	1.99	85.3	8.1	74.8	12.0
1pky :A	25.9	2.8	18.4	1.12	77.5	0.6	69.0	0.9	27.8	5.3	20.3	1.23	78.5	1.0	70.2	1.7
1poy :1	29.7	11.5	26.2	1.64	84.0	2.2	75.3	3.7	29.7	10.2	24.4	1.53	82.5	2.0	74.0	3.3
1qor :A	37.5	18.0	45.7	1.92	86.1	5.6	74.6	8.5	37.5	19.3	48.8	2.06	87.8	6.0	75.8	9.1
1rah :B	31.3	10.0	53.6	1.47	84.5	5.7	65.2	7.3	22.9	3.2	42.3	1.16	82.1	1.8	60.6	2.3
1scu :D	44.3	20.4	74.5	1.86	89.8	13.7	71.6	16.4	46.8	22.5	77.1	1.92	90.7	15.0	73.1	18.0
1scu :E	37.0	15.3	46.9	1.71	84.1	5.8	71.2	8.4	39.5	16.1	46.4	1.69	82.7	6.1	70.8	8.8
1set :A	40.0	18.1	41.1	1.82	83.3	5.3	73.6	8.1	42.7	19.8	42.1	1.87	82.9	5.8	73.9	8.9
1sft :A	41.0	19.6	61.8	1.92	88.0	9.3	72.9	12.6	39.8	17.3	56.9	1.77	85.7	8.2	70.9	11.1
1tph :1	54.8	33.1	62.2	2.53	89.1	10.8	80.7	16.3	52.4	30.2	57.9	2.36	87.6	9.8	78.9	14.8
1xik :A	25.8	10.2	43.2	1.65	88.0	3.6	71.7	5.3	19.4	5.0	35.3	1.35	87.4	1.8	69.6	2.6
2cst :A	32.5	12.1	45.0	1.59	84.4	4.8	69.7	6.8	31.3	10.6	42.6	1.51	83.4	4.2	68.7	6.0
2eip :A	18.8	-3.5	10.3	0.84	77.2	-0.5	70.0	-0.9	25.0	2.7	13.8	1.12	78.1	0.4	71.5	0.7
2hhm :A	39.0	19.3	42.1	1.98	85.5	5.2	75.6	8.2	41.5	20.2	41.5	1.95	84.2	5.5	75.1	8.6
2pcd :B	25.9	5.3	66.7	1.25	85.3	6.0	53.8	5.6	24.7	4.1	63.6	1.2	84.0	4.6	52.5	4.3
2pcd :N	19.4	1.4	79.4	1.08	86.0	4.0	37.0	2.1	20.9	2.3	82.9	1.13	88.0	6.5	38.6	3.4
2pol :A	49.2	24.5	45.6	1.99	82.5	7.3	74.9	11.2	46.0	20.9	42.0	1.83	81.1	6.2	73.1	9.6
3lad :A	39.1	16.9	54.2	1.76	85.3	7.5	71.0	10.4	44.3	20.8	58.0	1.88	85.7	9.3	72.9	12.8
3mde :A	48.1	23.6	53.6	1.96	84.4	8.9	74.5	12.9	51.9	26.4	55.6	2.03	84.4	9.9	75.5	14.4
6gsv :A	46.5	23.2	52.6	2	85.0	8.3	74.8	12.2	39.5	16.2	44.7	1.7	82.5	5.8	71.2	8.6
8cat :A	39.2	17.4	53.4	1.8	85.6	7.3	71.9	10.3	34.2	11.9	45.6	1.54	82.8	5.0	68.4	7.1
1gp1 :A	28.6	13.1	28.6	1.85	87.0	2.4	77.9	4.1	0.0	0.0	0.0	0	100.0	0.0	86.8	0.0

TAB. 1 – Comparison of ET and JET traces results for homodimers of the Huang benchmark. Protein `1gp1 :A` has been evaluated because it belongs to the dataset but its performance has not been included in the average (denoted `All`) reported in the article because no answer is obtained with ET. Structure `1rvv` in the Huang dataset has been replaced by structure `1nqv` which contains only 5 chains instead of 30 ; this change makes no difference in JET/ET prediction.

Heterodimers																
	**JET trace results**								**ET trace results**							
pdbCode	Sen	ScSen	PPV	ScPPV	Spe	ScSpe	Acc	ScAcc	Sen	ScSen	PPV	ScPPV	Spe	ScSpe	Acc	ScAcc
1all :A	42.2	17.0	57.6	1.68	83.7	8.9	69.5	11.7	42.2	18.6	61.3	1.78	86.0	9.7	71.0	12.7
1all :B	48.9	24.4	66.7	2	87.8	12.2	74.8	16.3	40.0	14.8	52.9	1.59	82.2	7.4	68.1	9.9
1hcg :A	26.9	10.7	25.9	1.66	85.7	2.0	76.5	3.3	19.2	3.0	18.5	1.18	84.3	0.5	74.1	0.9
1hcg :B	31.6	6.1	46.2	1.24	78.1	3.6	60.8	4.5	26.3	0.8	38.5	1.03	75.0	0.5	56.9	0.6
1luc :A	37.8	16.7	33.3	1.79	82.7	3.8	74.4	6.2	46.7	26.8	43.8	2.35	86.3	6.1	78.9	10.0
1luc :B	50.0	29.0	48.9	2.38	86.5	7.5	79.0	11.9	47.8	28.2	50.0	2.43	87.6	7.3	79.5	11.6
1scu :D	44.3	20.4	74.5	1.86	89.8	13.7	71.6	16.4	46.8	22.5	77.1	1.92	90.7	15.0	73.1	18.0
1scu :E	37.0	15.3	46.9	1.71	84.1	5.8	71.2	8.4	39.5	16.1	46.4	1.69	82.7	6.1	70.8	8.8
1tco :A	17.3	2.1	37.1	1.14	85.8	1.0	63.5	1.4	17.3	3.0	39.4	1.21	87.1	1.4	64.3	1.9
1tco :B	26.7	5.9	51.6	1.28	83.1	4.0	60.4	4.7	35.0	12.9	63.6	1.58	86.5	8.7	65.8	10.3
1tco :C	31.0	9.7	47.4	1.45	83.3	4.7	66.3	6.3	31.0	10.8	50.0	1.53	85.0	5.2	67.4	7.0
1tcr :A	34.6	11.8	45.0	1.51	82.1	5.0	68.0	7.0	42.3	19.5	55.0	1.85	85.4	8.2	72.6	11.6
1tcr :B	37.7	17.6	51.3	1.88	86.5	6.6	73.2	9.6	32.1	11.5	42.5	1.56	83.7	4.3	69.6	6.3
1ubs :A	55.6	31.9	45.5	2.35	84.0	7.7	78.5	12.3	61.1	36.9	48.9	2.53	84.7	8.9	80.1	14.3
1ubs :B	39.2	21.3	45.5	2.19	87.7	5.6	77.6	8.8	43.1	24.4	47.8	2.31	87.7	6.4	78.5	10.1
1wdc :A	24.1	-0.9	81.3	0.96	70.0	-5.0	31.3	-1.6	24.1	-0.9	81.3	0.96	70.0	-5.0	31.3	-1.6
1wdc :B	36.0	13.3	60.0	1.58	85.4	8.1	66.7	10.1	40.0	15.8	62.5	1.65	85.4	9.6	68.2	11.9
1wdc :C	40.9	16.8	56.3	1.7	84.3	8.3	69.9	11.1	47.7	22.9	63.6	1.92	86.5	11.3	73.7	15.2
2pcd :B	25.9	5.3	66.7	1.25	85.3	6.0	53.8	5.6	24.7	4.1	63.6	1.2	84.0	4.6	52.5	4.3
2pcd :N	19.4	1.4	79.4	1.08	86.0	4.0	37.0	2.1	20.9	2.3	82.9	1.13	88.0	6.5	38.6	3.4
8atc :A	37.1	18.9	34.2	2.03	85.5	3.8	77.4	6.4	37.1	19.4	35.1	2.09	86.1	3.9	77.9	6.5
8atc :B	37.3	16.8	73.1	1.82	90.8	11.3	69.3	13.5	35.3	15.6	72.0	1.79	90.8	10.5	68.5	12.5
9atc :A	42.1	22.8	39.0	2.18	85.6	5.0	77.8	8.2	39.5	21.1	38.5	2.15	86.2	4.6	77.8	7.6
9atc :B	36.5	13.8	63.3	1.61	86.3	9.0	66.7	10.9	36.5	13.8	63.3	1.61	86.3	9.0	66.7	10.9

TAB. 2 – Comparison of ET and JET traces results for heterodimers of the Huang benchmark

Transients																
	**JET trace results**								**ET trace results**							
pdbCode	Sen	ScSen	PPV	ScPPV	Spe	ScSpe	Acc	ScAcc	Sen	ScSen	PPV	ScPPV	Spe	ScSpe	Acc	ScAcc
1apm :E	52.2	33.4	50.0	2.78	88.6	7.3	82.0	12.0	60.9	41.3	56.0	3.12	89.5	9.1	84.4	14.9
1efu :A	31.2	7.3	37.5	1.31	79.1	2.9	65.3	4.2	37.7	11.9	42.0	1.46	79.1	4.8	67.2	6.8
1efu :B	45.2	21.9	60.0	1.94	86.5	9.8	73.7	13.5	53.4	29.7	69.6	2.25	89.6	13.3	78.4	18.4
1g3n :A	15.8	-4.8	33.3	0.77	75.7	-3.6	49.8	-4.1	15.8	-5.2	32.7	0.75	75.0	-4.0	49.4	-4.5
1g3n :B	12.5	-4.2	15.0	0.75	82.3	-1.0	68.3	-1.7	4.2	-15.0	4.3	0.22	77.1	-3.8	62.5	-6.0
1g3n :C	27.0	6.1	27.8	1.29	80.7	1.7	69.2	2.6	16.2	-3.5	17.6	0.82	79.3	-1.0	65.7	-1.5
1got :A	44.2	21.4	30.2	1.94	81.1	3.9	75.4	6.7	51.2	29.1	36.1	2.31	83.3	5.4	78.3	9.1
1got :B	20.2	1.3	39.6	1.07	81.9	0.8	59.1	1.0	20.2	2.1	41.3	1.12	83.1	1.2	59.8	1.6
1got :G	30.2	4.4	86.7	1.17	86.7	12.5	44.8	6.5	32.6	6.7	93.3	1.26	93.3	19.2	48.3	9.9
1k9o :E	36.0	22.3	40.9	2.63	90.4	4.1	82.0	6.9	32.0	19.6	40.0	2.58	91.2	3.6	82.0	6.1
1k9o :I	12.5	-3.7	4.7	0.77	83.5	-0.2	79.2	-0.5	12.5	-5.6	4.2	0.69	81.5	-0.4	77.4	-0.7
1rrp :A	20.0	-1.7	51.4	0.92	76.0	-2.2	44.7	-1.9	26.7	4.3	66.7	1.19	83.1	5.5	51.6	4.8
1rrp :B	32.7	12.0	73.9	1.58	89.8	10.6	63.1	11.2	30.8	11.0	72.7	1.55	89.8	9.6	62.2	10.3
1rrp :C	22.4	0.6	56.7	1.03	79.0	0.8	47.8	0.7	28.9	5.8	68.8	1.25	83.9	7.0	53.6	6.3
1ugh :E	44.8	31.2	59.1	3.28	93.2	6.8	84.5	11.2	55.2	39.0	61.5	3.42	92.4	8.6	85.7	14.1
1ugh :I	11.1	-9.2	21.4	0.55	73.8	-5.9	49.3	-7.2	0.0	0.0	0.0	0	100.0	0.0	60.9	0.0
1ytf :A	48.2	22.3	75.0	1.86	89.2	15.1	72.7	18.0	50.0	24.1	77.8	1.93	90.4	16.3	74.1	19.4
1ytf :B	27.3	1.2	100.0	1.05	100.0	26.1	30.4	2.3	22.7	-3.4	83.3	0.87	0.0	-73.9	21.7	-6.4
1ytf :D	26.5	2.0	75.0	1.08	80.0	4.5	42.9	2.7	27.9	3.5	79.2	1.14	83.3	7.8	44.9	4.8

TAB. 3 – Comparison of ET and JET traces results for transients of the Huang benchmark

Homodimers																
	**JET with clustering**								**ET with clustering**							
pdbCode	Sen	ScSen	PPV	ScPPV	Spe	ScSpe	Acc	ScAcc	Sen	ScSen	PPV	ScPPV	Spe	ScSpe	Acc	ScAcc
1bnc :A	19.4	8.4	41.2	1.76	91.5	2.6	74.7	3.9	30.6	9.1	33.3	1.43	81.4	2.8	69.5	4.3
1daa :A	48.5	32.0	91.4	2.94	97.9	14.5	82.5	19.9	51.5	31.7	81.0	2.6	94.5	14.3	81.1	19.7
1dpg :A	21.4	11.1	48.6	2.08	93.1	3.4	76.3	5.2	36.9	16.6	42.5	1.81	84.7	5.1	73.5	7.8
1ecp :B	43.9	21.3	94.7	1.94	97.7	20.3	71.4	20.8	37.8	17.0	88.6	1.81	95.3	16.2	67.3	16.6
1efu :A	35.1	19.4	64.3	2.24	92.1	7.8	75.7	11.1	37.7	12.3	42.6	1.48	79.6	5.0	67.5	7.1
1efu :B	26.0	12.9	61.3	1.98	92.6	5.8	72.0	8.0	52.1	28.7	69.1	2.23	89.6	12.9	78.0	17.8
1frp :A	30.6	15.9	78.8	2.08	95.0	9.7	70.5	12.0	35.3	16.5	71.4	1.88	91.4	10.1	70.1	12.6
1fuq :A	55.6	37.7	50.8	3.12	89.5	7.4	84.0	12.3	66.7	42.5	45.0	2.76	84.1	8.3	81.3	13.9
1gdh :A	18.8	3.9	36.1	1.26	86.6	1.6	67.2	2.2	23.2	4.5	35.6	1.24	83.1	1.8	66.0	2.6
1ges :A	32.2	19.3	84.1	2.5	96.9	9.8	75.1	13.0	46.1	23.3	67.9	2.02	89.0	11.8	74.6	15.7
1glq :A	67.4	45.7	82.9	3.1	94.9	16.7	87.6	24.4	37.2	16.1	47.1	1.76	84.7	5.9	72.0	8.6
1gpm :B	19.8	7.8	38.6	1.66	90.5	2.4	74.0	3.7	32.6	9.5	32.9	1.41	79.9	2.9	68.8	4.4
1hur :A	54.5	29.5	72.7	2.18	89.8	14.8	78.0	19.7	40.9	22.0	72.0	2.16	92.0	11.0	75.0	14.6
1hyh :A	18.8	5.5	68.8	1.41	91.9	5.2	56.3	5.3	27.4	4.0	57.1	1.17	80.5	3.8	54.6	3.9
1ids :A	42.4	16.6	68.3	1.65	86.0	11.8	67.9	13.8	22.7	3.2	48.4	1.17	82.8	2.3	57.9	2.7
1ies :B	25.0	3.9	67.9	1.19	84.2	5.3	50.4	4.5	22.4	4.3	70.8	1.24	87.7	5.8	50.4	4.9
1leh :A	0.0	-13.3	0.0	0	85.2	-1.5	76.8	-2.6	3.7	-15.1	2.0	0.2	79.5	-1.7	72.0	-3.0
1mas :A	57.7	32.8	58.8	2.32	86.3	11.2	79.0	16.6	26.9	11.3	43.8	1.72	88.2	3.8	72.7	5.7
1mld :A	44.1	27.5	70.3	2.67	93.3	9.9	80.4	14.5	52.5	29.8	60.8	2.31	87.9	10.6	78.6	15.7
1nhk :L	11.1	-10.7	16.7	0.51	73.0	-5.2	52.7	-7.0	33.3	10.6	48.0	1.47	82.4	5.2	66.4	6.9
1nqv :A	49.3	18.8	94.3	1.62	95.8	26.3	68.7	21.9	29.9	7.2	76.9	1.32	87.5	10.1	53.9	8.4
1oro :A	51.1	28.3	62.2	2.24	88.0	10.9	77.8	15.7	53.3	28.0	58.5	2.11	85.5	10.8	76.5	15.6
1osj :A	25.8	10.6	44.7	1.69	88.6	3.8	72.0	5.6	43.9	21.5	51.8	1.96	85.3	7.7	74.4	11.4
1pky :A	18.5	5.8	23.8	1.45	88.4	1.1	76.9	1.9	27.8	8.0	23.1	1.41	81.8	1.6	72.9	2.6
1poy :1	21.6	10.8	32.0	2	91.2	2.1	80.1	3.5	27.0	9.7	25.0	1.56	84.5	1.9	75.3	3.1
1qor :A	50.0	35.6	82.4	3.47	96.7	11.1	85.6	16.9	37.5	21.4	55.3	2.33	90.6	6.7	78.0	10.2
1rah :B	43.8	15.7	56.8	1.56	81.0	9.0	67.4	11.4	20.8	2.7	41.7	1.15	83.3	1.5	60.6	1.9
1scu :D	50.6	29.3	95.2	2.37	98.3	19.6	79.2	23.5	45.6	22.7	80.0	1.99	92.4	15.2	73.6	18.2
1scu :E	27.2	11.9	48.9	1.78	89.3	4.5	72.2	6.5	37.0	16.0	48.4	1.76	85.0	6.1	71.9	8.8
1set :A	28.0	14.8	47.7	2.12	91.1	4.3	76.9	6.7	40.0	19.0	42.9	1.9	84.5	5.5	74.5	8.5
1sft :A	48.2	30.0	85.1	2.65	96.0	14.2	80.6	19.3	39.8	20.0	64.7	2.01	89.7	9.5	73.6	12.9
1tph :1	54.8	35.5	69.7	2.84	92.2	11.5	83.0	17.4	50.0	28.9	58.3	2.37	88.4	9.4	78.9	14.2
1xik :A	37.1	19.8	56.1	2.14	89.7	7.0	75.9	10.4	19.4	5.9	37.5	1.43	88.6	2.1	70.5	3.1
2cst :A	28.9	14.3	55.8	1.98	91.0	5.6	73.5	8.1	28.9	9.2	41.4	1.47	83.9	3.6	68.4	5.2
2eip :A	18.8	-5.1	9.7	0.79	75.4	-0.7	68.5	-1.3	25.0	4.2	14.8	1.2	79.8	0.6	73.1	1.0
2hhm :A	9.8	2.0	26.7	1.26	92.8	0.5	75.1	0.8	41.5	22.3	45.9	2.16	86.8	6.0	77.2	9.5
2pcd :B	37.6	12.0	78.0	1.47	88.0	13.6	61.3	12.8	24.7	4.7	65.6	1.24	85.3	5.3	53.1	5.0
2pcd :N	26.6	2.8	82.2	1.12	84.0	7.8	41.8	4.1	18.7	2.3	83.9	1.14	90.0	6.4	37.6	3.4
2pol :A	23.8	7.4	33.3	1.46	85.8	2.2	71.6	3.4	46.0	21.7	43.3	1.89	82.1	6.4	73.8	9.9
3lad :A	28.7	17.7	80.5	2.61	96.9	7.9	75.9	10.9	41.7	20.0	59.3	1.92	87.2	8.9	73.2	12.3
3mde :A	53.2	37.3	91.1	3.34	98.0	14.0	85.8	20.4	51.9	28.5	60.6	2.22	87.3	10.7	77.7	15.6
6gsv :A	53.5	33.2	69.7	2.64	91.7	11.9	81.6	17.5	37.2	16.4	47.1	1.78	85.0	5.9	72.4	8.6
8cat :A	35.0	21.2	75.0	2.53	95.1	8.9	77.3	12.5	31.7	11.4	46.3	1.56	84.6	4.8	68.9	6.8
1gp1 :A	61.9	41.3	46.4	3.01	87.0	7.5	83.1	12.8	0.0	0.0	0.0	0	100.0	0.0	86.8	0.0

TAB. 4 – Comparison of ET and JET performance after clustering for homodimers of the Huang benchmark

Heterodimers																
	**JET with clustering**								**ET with clustering**							
pdbCode	Sen	ScSen	PPV	ScPPV	Spe	ScSpe	Acc	ScAcc	Sen	ScSen	PPV	ScPPV	Spe	ScSpe	Acc	ScAcc
1all :A	37.8	12.6	51.5	1.5	81.4	6.6	66.4	8.6	42.2	20.1	65.5	1.91	88.4	10.5	72.5	13.8
1all :B	51.1	20.7	56.1	1.68	80.0	10.4	70.4	13.8	40.0	15.6	54.5	1.64	83.3	7.8	68.9	10.4
1hcg :A	30.8	7.3	20.5	1.31	77.9	1.3	70.5	2.3	19.2	3.0	18.5	1.18	84.3	0.5	74.1	0.9
1hcg :B	68.4	11.6	44.8	1.2	50.0	6.9	56.9	8.6	26.3	4.7	45.5	1.22	81.3	2.8	60.8	3.5
1luc :A	44.4	27.1	47.6	2.56	88.8	6.2	80.6	10.1	46.7	26.8	43.8	2.35	86.3	6.1	78.9	10.0
1luc :B	60.9	43.9	73.7	3.59	94.4	11.3	87.5	18.0	47.8	29.5	53.7	2.61	89.3	7.6	80.8	12.1
1scu :D	50.6	29.3	95.2	2.37	98.3	19.6	79.2	23.5	45.6	22.7	80.0	1.99	92.4	15.2	73.6	18.2
1scu :E	27.2	11.9	48.9	1.78	89.3	4.5	72.2	6.5	37.0	16.0	48.4	1.76	85.0	6.1	71.9	8.8
1tco :A	21.3	5.7	44.4	1.36	87.1	2.7	65.7	3.7	14.7	1.6	36.7	1.12	87.7	0.8	63.9	1.1
1tco :B	45.0	18.2	67.5	1.68	85.4	12.2	69.1	14.6	31.7	10.9	61.3	1.52	86.5	7.3	64.4	8.7
1tco :C	55.2	14.7	44.4	1.36	66.7	7.1	62.9	9.6	31.0	13.1	56.3	1.73	88.3	6.3	69.7	8.5
1tcr :A	46.2	19.9	52.2	1.76	82.1	8.4	71.4	11.8	40.4	19.2	56.8	1.91	87.0	8.1	73.1	11.4
1tcr :B	45.3	19.0	47.1	1.72	80.9	7.1	71.1	10.4	32.1	11.5	42.5	1.56	83.7	4.3	69.6	6.3
1ubs :A	44.4	31.5	66.7	3.44	94.7	7.6	84.9	12.2	61.1	38.0	51.2	2.64	86.0	9.1	81.2	14.7
1ubs :B	3.9	3.1	100.0	4.82	100.0	0.8	80.1	1.3	39.2	21.7	46.5	2.24	88.2	5.7	78.0	9.0
1wdc :A	40.7	1.7	88.0	1.04	70.0	9.1	45.3	2.8	24.1	-0.9	81.3	0.96	70.0	-5.0	31.3	-1.6
1wdc :B	30.0	9.5	55.6	1.47	85.4	5.8	64.4	7.2	36.0	13.3	60.0	1.58	85.4	8.1	66.7	10.1
1wdc :C	47.7	18.4	53.8	1.63	79.8	9.1	69.2	12.2	47.7	22.9	63.6	1.92	86.5	11.3	73.7	15.2
2pcd :B	37.6	12.0	78.0	1.47	88.0	13.6	61.3	12.8	24.7	4.7	65.6	1.24	85.3	5.3	53.1	5.0
2pcd :N	26.6	2.8	82.2	1.12	84.0	7.8	41.8	4.1	18.7	2.3	83.9	1.14	90.0	6.4	37.6	3.4
8atc :A	34.3	21.3	44.4	2.64	91.3	4.3	81.7	7.2	37.1	20.3	37.1	2.21	87.3	4.1	78.8	6.8
8atc :B	58.8	27.3	75.0	1.87	86.8	18.3	75.6	21.9	35.3	17.2	78.3	1.95	93.4	11.5	70.1	13.8
9atc :A	39.5	23.9	45.5	2.54	89.7	5.2	80.7	8.6	39.5	22.0	40.5	2.26	87.4	4.8	78.8	7.9
9atc :B	53.8	25.8	75.7	1.92	88.8	16.8	75.0	20.3	34.6	13.4	64.3	1.63	87.5	8.7	66.7	10.6

Tab. 5 – Comparison of ET and JET performance after clustering for heterodimers of the Huang benchmark

Transients																
	**JET with clustering**								**ET with clustering**							
pdbCode	Sen	ScSen	PPV	ScPPV	Spe	ScSpe	Acc	ScAcc	Sen	ScSen	PPV	ScPPV	Spe	ScSpe	Acc	ScAcc
1apm :E	52.2	41.2	85.7	4.77	98.1	9.0	89.8	14.8	58.7	39.9	56.3	3.13	90.0	8.8	84.4	14.4
1efu :A	35.1	19.4	64.3	2.24	92.1	7.8	75.7	11.1	37.7	12.3	42.6	1.48	79.6	5.0	67.5	7.1
1efu :B	26.0	12.9	61.3	1.98	92.6	5.8	72.0	8.0	52.1	28.7	69.1	2.23	89.6	12.9	78.0	17.8
1g3n :A	15.8	0.0	43.2	1	84.1	0.0	54.5	0.0	15.8	-4.8	33.3	0.77	75.7	-3.6	49.8	-4.1
1g3n :B	12.5	-21.7	7.3	0.37	60.4	-5.4	50.8	-8.7	4.2	-14.2	4.5	0.23	78.1	-3.5	63.3	-5.7
1g3n :C	40.5	17.9	38.5	1.79	82.2	4.9	73.3	7.7	16.2	-2.4	18.8	0.87	80.7	-0.7	66.9	-1.0
1got :A	44.2	32.2	57.6	3.7	94.0	5.9	86.2	10.0	48.8	28.5	37.5	2.41	85.0	5.3	79.3	8.9
1got :B	17.0	6.0	57.1	1.54	92.5	3.5	64.6	4.4	18.1	3.1	44.7	1.21	86.9	1.8	61.4	2.3
1got :G	74.4	10.6	86.5	1.17	66.7	30.5	72.4	15.8	30.2	6.1	92.9	1.25	93.3	17.5	46.6	9.0
1k9o :E	52.0	37.7	56.5	3.64	92.6	6.9	86.3	11.7	32.0	20.8	44.4	2.86	92.6	3.8	83.2	6.5
1k9o :I	18.8	3.7	7.5	1.24	85.1	0.2	81.1	0.4	12.5	-3.7	4.7	0.77	83.5	-0.2	79.2	-0.5
1rrp :A	24.4	0.2	56.4	1.01	76.0	0.3	47.2	0.2	26.7	4.9	68.6	1.23	84.5	6.2	52.2	5.5
1rrp :B	46.2	19.1	80.0	1.71	89.8	16.9	69.4	17.9	30.8	11.0	72.7	1.55	89.8	9.6	62.2	10.3
1rrp :C	25.0	0.4	55.9	1.01	75.8	0.4	47.8	0.4	28.9	5.8	68.8	1.25	83.9	7.0	53.6	6.3
1ugh :E	75.9	51.0	55.0	3.05	86.4	11.2	84.5	18.4	55.2	39.6	64.0	3.55	93.2	8.7	86.3	14.3
1ugh :I	29.6	-18.2	24.2	0.62	40.5	-11.7	36.2	-14.2	0.0	0.0	0.0	0	100.0	0.0	60.9	0.0
1ytf :A	57.1	29.8	84.2	2.09	92.8	20.1	78.4	24.0	50.0	24.8	80.0	1.99	91.6	16.7	74.8	20.0
1ytf :B	54.5	-2.0	92.3	0.97	0.0	-43.5	52.2	-3.8	20.4	-3.5	81.8	0.86	0.0	-76.1	19.6	-6.6
1ytf :D	38.2	4.6	78.8	1.14	76.7	10.3	50.0	6.3	26.5	3.0	78.3	1.13	83.3	6.8	43.9	4.2

Tab. 6 – Comparison of ET and JET performance after clustering for transients of the Huang benchmark

**Homodimers**																
	**JET with clustering - conservation+PC properties**								**iJET - conservation+PC properties**							
pdbCode	Sen	ScSen	PPV	ScPPV	Spe	ScSpe	Acc	ScAcc	Sen	ScSen	PPV	ScPPV	Spe	ScSpe	Acc	ScAcc
1bnc :A	22.2	7.6	35.6	1.52	87.7	2.3	72.4	3.5	22.2	7.6	35.6	1.52	87.7	2.3	72.4	3.5
1daa :A	57.6	39.2	97.4	3.13	99.3	17.7	86.3	24.4	57.6	39.2	97.4	3.13	99.3	17.7	86.3	24.4
1dpg :A	35.7	20.7	55.6	2.37	91.3	6.3	78.3	9.7	33.3	19.4	56.0	2.39	92.0	5.9	78.3	9.1
1ecp :B	36.6	18.1	96.8	1.98	98.8	17.3	68.5	17.7	34.1	16.9	96.6	1.98	98.8	16.1	67.3	16.5
1efu :A	28.6	17.4	73.3	2.55	95.8	7.0	76.5	10.0	26.0	15.9	74.1	2.58	96.3	6.4	76.1	9.1
1efu :B	31.5	16.3	63.9	2.07	92.0	7.3	73.3	10.1	30.1	16.6	68.8	2.22	93.9	7.4	74.2	10.3
1frp :A	34.1	18.5	82.9	2.18	95.7	11.3	72.3	14.0	32.9	18.2	84.8	2.24	96.4	11.1	72.3	13.8
1fuq :A	61.1	40.6	48.5	2.97	87.4	7.9	83.1	13.2	55.6	37.4	50.0	3.06	89.2	7.3	83.7	12.2
1gdh :A	37.7	18.6	56.5	1.97	88.4	7.5	73.9	10.6	29.0	13.6	54.1	1.89	90.1	5.5	72.6	7.8
1ges :A	32.2	18.7	80.4	2.39	96.0	9.5	74.6	12.6	26.1	15.3	81.1	2.41	96.9	7.7	73.1	10.3
1glq :A	62.8	38.6	69.2	2.59	89.8	14.1	82.6	20.6	53.5	36.7	85.2	3.19	96.6	13.4	85.1	19.6
1gpm :B	22.1	7.5	35.2	1.51	87.6	2.3	72.4	3.5	20.9	7.7	36.7	1.58	89.0	2.3	73.2	3.6
1hur :A	63.6	28.0	59.6	1.79	78.4	14.0	73.5	18.7	63.6	29.5	62.2	1.87	80.7	14.8	75.0	19.7
1hyh :A	14.5	1.6	54.8	1.12	88.6	1.5	52.5	1.6	15.4	2.5	58.1	1.19	89.4	2.3	53.3	2.4
1ids :A	31.8	16.1	84.0	2.02	95.7	11.4	69.2	13.4	31.8	15.5	80.8	1.95	94.6	11.0	68.6	12.8
1ies :B	34.2	7.1	72.2	1.26	82.5	9.5	54.9	8.2	35.5	8.5	75.0	1.31	84.2	11.3	56.4	9.7
1leh :A	37.0	14.9	16.7	1.67	79.5	1.6	75.3	3.0	3.7	-12.9	2.2	0.22	82.0	-1.4	74.2	-2.6
1mas :A	42.3	23.3	56.4	2.22	88.9	7.9	77.1	11.8	40.4	22.3	56.8	2.24	89.5	7.6	77.1	11.3
1mld :A	47.5	31.4	77.8	2.95	95.2	11.2	82.6	16.5	37.3	24.8	78.6	2.98	96.4	8.9	80.8	13.1
1nhk :L	38.9	2.5	35.0	1.07	64.9	1.2	56.4	1.7	44.4	9.9	42.1	1.29	70.3	4.8	61.8	6.5
1nqv :A	38.8	12.7	86.7	1.49	91.7	17.8	60.9	14.8	41.8	14.0	87.5	1.5	91.7	19.5	62.6	16.3
1oro :A	60.0	31.0	57.4	2.07	82.9	11.9	76.5	17.2	57.8	31.9	61.9	2.23	86.3	12.2	78.4	17.7
1osj :A	33.3	15.3	48.9	1.85	87.5	5.5	73.2	8.1	31.8	15.0	50.0	1.89	88.6	5.4	73.6	7.9
1pky :A	18.5	3.6	20.4	1.24	85.8	0.7	74.8	1.2	18.5	3.3	20.0	1.22	85.5	0.7	74.5	1.1
1poy :1	18.9	5.9	23.3	1.46	88.1	1.1	77.1	1.9	18.9	7.2	25.9	1.62	89.7	1.4	78.4	2.3
1qor :A	41.1	27.9	74.2	3.13	95.6	8.7	82.6	13.3	42.9	29.3	75.0	3.16	95.6	9.1	83.1	13.9
1rah :B	45.8	8.7	44.9	1.23	67.9	5.0	59.8	6.3	39.6	10.8	50.0	1.38	77.4	6.2	63.6	7.9
1scu :D	51.9	30.1	95.3	2.38	98.3	20.1	79.7	24.1	53.2	31.3	97.7	2.44	99.2	21.0	80.7	25.1
1scu :E	30.9	13.9	50.0	1.82	88.3	5.3	72.5	7.6	28.4	12.5	48.9	1.78	88.8	4.7	72.2	6.8
1set :A	30.7	18.4	56.1	2.49	93.0	5.3	79.0	8.3	30.7	19.6	62.2	2.76	94.6	5.7	80.2	8.8
1sft :A	51.8	31.3	81.1	2.52	94.3	14.8	80.6	20.1	53.0	34.4	91.7	2.85	97.7	16.3	83.3	22.1
1tph :1	71.4	46.3	69.8	2.84	89.9	15.1	85.4	22.7	69.0	45.1	70.7	2.88	90.7	14.7	85.4	22.1
1xik :A	33.9	19.9	63.6	2.43	93.1	7.1	77.6	10.4	32.3	18.8	62.5	2.39	93.1	6.6	77.2	9.8
2cst :A	16.9	7.3	50.0	1.77	93.4	2.9	71.8	4.1	16.9	8.7	58.3	2.07	95.3	3.4	73.1	4.9
2eip :A	56.3	26.3	23.1	1.87	73.7	3.7	71.5	6.5	50.0	22.3	22.2	1.81	75.4	3.1	72.3	5.5
2hhm :A	7.3	0.1	21.4	1.01	92.8	0.0	74.6	0.0	7.3	0.6	23.1	1.09	93.4	0.2	75.1	0.2
2pcd :B	42.4	13.6	78.3	1.47	86.7	15.4	63.1	14.5	41.2	17.4	92.1	1.73	96.0	19.8	66.9	18.5
2pcd :N	20.1	3.7	90.3	1.23	94.0	10.4	39.7	5.5	20.1	3.2	87.5	1.19	92.0	8.9	39.2	4.7
2pol :A	30.2	12.3	38.8	1.69	85.8	3.7	73.1	5.7	28.6	12.6	40.9	1.79	87.7	3.7	74.2	5.8
3lad :A	38.3	23.2	78.6	2.55	95.3	10.4	77.7	14.3	39.1	25.2	86.5	2.81	97.3	11.2	79.4	15.5
3mde :A	46.8	31.9	85.7	3.14	97.1	12.0	83.3	17.4	46.8	31.9	85.7	3.14	97.1	12.0	83.3	17.4
6gsv :A	51.2	31.5	68.8	2.61	91.7	11.3	81.0	16.6	48.8	31.0	72.4	2.74	93.3	11.1	81.6	16.4
8cat :A	21.7	13.0	74.3	2.51	96.8	5.5	74.6	7.7	19.2	11.5	74.2	2.5	97.2	4.8	74.1	6.8
1gp1 :A	57.1	31.4	34.3	2.22	80.0	5.7	76.5	9.7	61.9	35.4	36.1	2.34	80.0	6.5	77.2	10.9

TAB. 7 – Left : JET with clustering based on conservation and physical-chemical properties (PC). Right : iJET with $i = 10$ where selection of clustered residues is obtained by a consensus of 7 distinguished runs. The proteins listed are all the homodimers in the Huang benchmark.

Heterodimers																
	**JET with clustering - conservation+PC properties**								**iJET - conservation+PC properties**							
pdbCode	Sen	ScSen	PPV	ScPPV	Spe	ScSpe	Acc	ScAcc	Sen	ScSen	PPV	ScPPV	Spe	ScSpe	Acc	ScAcc
1all :A	46.7	19.9	60.0	1.75	83.7	10.4	71.0	13.7	44.4	20.8	64.5	1.88	87.2	10.9	72.5	14.3
1all :B	66.7	35.6	71.4	2.14	86.7	17.8	80.0	23.7	62.2	32.6	70.0	2.1	86.7	16.3	78.5	21.7
1hcg :A	57.7	31.8	34.9	2.23	80.0	5.9	76.5	10.0	38.5	16.2	27.0	1.73	80.7	3.0	74.1	5.1
1hcg :B	78.9	22.1	51.7	1.39	56.3	13.1	64.7	16.5	78.9	22.1	51.7	1.39	56.3	13.1	64.7	16.5
1luc :A	48.9	30.3	48.9	2.63	88.3	6.9	81.0	11.3	46.7	28.9	48.8	2.63	88.8	6.6	81.0	10.7
1luc :B	67.4	46.4	66.0	3.21	91.0	12.0	86.2	19.1	65.2	44.2	63.8	3.11	90.4	11.4	85.3	18.2
1scu :D	51.9	30.1	95.3	2.38	98.3	20.1	79.7	24.1	53.2	31.3	97.7	2.44	99.2	21.0	80.7	25.1
1scu :E	30.9	13.9	50.0	1.82	88.3	5.3	72.5	7.6	28.4	12.5	48.9	1.78	88.8	4.7	72.2	6.8
1tco :A	33.3	14.2	56.8	1.74	87.7	6.9	70.0	9.3	26.7	11.0	55.6	1.7	89.7	5.3	69.1	7.2
1tco :B	50.0	23.2	75.0	1.86	88.8	15.6	73.2	18.6	43.3	21.9	81.3	2.02	93.3	14.7	73.2	17.6
1tco :C	58.6	14.8	43.6	1.34	63.3	7.1	61.8	9.6	58.6	17.0	45.9	1.41	66.7	8.2	64.0	11.1
1tcr :A	34.6	16.9	58.1	1.95	89.4	7.1	73.1	10.0	36.5	18.8	61.3	2.06	90.2	8.0	74.3	11.2
1tcr :B	49.1	25.9	57.8	2.11	86.5	9.7	76.3	14.1	47.2	29.6	73.5	2.69	93.6	11.1	80.9	16.2
1ubs :A	58.3	41.7	67.7	3.5	93.3	10.0	86.6	16.1	61.1	43.9	68.8	3.55	93.3	10.5	87.1	17.0
1ubs :B	11.8	-2.1	17.6	0.85	85.6	-0.5	70.3	-0.9	11.8	2.0	25.0	1.21	90.8	0.5	74.4	0.8
1wdc :A	48.1	4.4	92.9	1.1	80.0	23.8	53.1	7.4	66.7	5.7	92.3	1.09	70.0	30.9	67.2	9.7
1wdc :B	58.0	20.9	59.2	1.56	75.6	12.7	68.9	15.8	58.0	25.4	67.4	1.78	82.9	15.5	73.5	19.3
1wdc :C	47.7	13.1	45.7	1.38	71.9	6.5	63.9	8.7	45.5	15.4	50.0	1.51	77.5	7.6	66.9	10.2
2pcd :B	42.4	13.6	78.3	1.47	86.7	15.4	63.1	14.5	41.2	17.4	92.1	1.73	96.0	19.8	66.9	18.5
2pcd :N	20.1	3.7	90.3	1.23	94.0	10.4	39.7	5.5	20.1	3.2	87.5	1.19	92.0	8.9	39.2	4.7
8atc :A	42.9	26.0	42.9	2.55	88.4	5.3	80.8	8.8	40.0	25.1	45.2	2.68	90.2	5.1	81.7	8.4
8atc :B	54.9	25.0	73.7	1.83	86.8	16.8	74.0	20.1	56.9	23.8	69.0	1.72	82.9	16.0	72.4	19.1
9atc :A	42.1	21.4	36.4	2.03	83.9	4.7	76.4	7.7	42.1	23.7	41.0	2.29	86.8	5.2	78.8	8.5
9atc :B	48.1	14.7	56.8	1.44	76.3	9.6	65.2	11.6	46.2	16.6	61.5	1.56	81.3	10.8	67.4	13.1

TAB. 8 – Left : JET with clustering based on conservation and physical-chemical properties (PC). Right : iJET with $i = 10$ where selection of clustered residues is obtained by a consensus of 7 distinguished runs. The proteins listed are all the heterodimers in the Huang benchmark

Transients																
	**JET with clustering - conservation+PC properties**								**iJET - conservation+PC properties**							
pdbCode	Sen	ScSen	PPV	ScPPV	Spe	ScSpe	Acc	ScAcc	Sen	ScSen	PPV	ScPPV	Spe	ScSpe	Acc	ScAcc
1apm :E	45.7	34.7	75.0	4.17	96.7	7.6	87.5	12.5	43.5	33.3	76.9	4.28	97.1	7.3	87.5	12.0
1efu :A	28.6	17.4	73.3	2.55	95.8	7.0	76.5	10.0	26.0	15.9	74.1	2.58	96.3	6.4	76.1	9.1
1efu :B	31.5	16.3	63.9	2.07	92.0	7.3	73.3	10.1	30.1	16.6	68.8	2.22	93.9	7.4	74.2	10.3
1g3n :A	11.9	-2.3	36.4	0.84	84.1	-1.7	52.8	-2.0	11.9	-2.3	36.4	0.84	84.1	-1.7	52.8	-2.0
1g3n :B	25.0	-15.0	12.5	0.63	56.3	-3.7	50.0	-6.0	20.8	-16.7	11.1	0.56	58.3	-4.2	50.8	-6.7
1g3n :C	37.8	19.8	45.2	2.1	87.4	5.4	76.7	8.5	32.4	16.2	42.9	1.99	88.1	4.4	76.2	6.9
1got :A	44.2	28.6	44.2	2.84	89.7	5.3	82.6	8.9	41.9	26.6	42.9	2.75	89.7	4.9	82.2	8.3
1got :B	26.6	6.5	49.0	1.32	83.8	3.8	62.6	4.8	21.3	3.5	44.4	1.2	84.4	2.1	61.0	2.6
1got :G	74.4	14.1	91.4	1.23	80.0	40.3	75.9	20.9	62.8	11.1	90.0	1.21	80.0	31.7	67.2	16.4
1k9o :E	64.0	43.5	48.5	3.12	87.5	8.0	83.9	13.5	60.0	44.5	60.0	3.86	92.6	8.2	87.6	13.8
1k9o :I	12.5	-5.6	4.2	0.69	81.5	-0.4	77.4	-0.7	18.8	1.8	6.7	1.1	83.1	0.1	79.2	0.2
1rrp :A	17.8	-0.2	55.2	0.99	81.7	-0.3	46.0	-0.3	16.7	-0.7	53.6	0.96	81.7	-0.9	45.3	-0.8
1rrp :B	53.8	21.4	77.8	1.66	86.4	18.9	71.2	20.1	51.9	24.0	87.1	1.86	93.2	21.1	73.9	22.5
1rrp :C	23.7	1.9	60.0	1.09	80.6	2.4	49.3	2.1	25.0	3.3	63.3	1.15	82.3	4.0	50.7	3.6
1ugh :E	75.9	51.6	56.4	3.13	87.1	11.3	85.1	18.6	75.9	53.5	61.1	3.39	89.4	11.8	87.0	19.3
1ugh :I	18.5	-17.7	20.0	0.51	52.4	-11.4	39.1	-13.9	22.2	4.8	50.0	1.28	85.7	3.1	60.9	3.8
1ytf :A	58.9	28.0	76.7	1.9	88.0	18.9	76.3	22.6	57.1	29.1	82.1	2.04	91.6	19.6	77.7	23.4
1ytf :B	65.9	-1.5	93.5	0.98	0.0	-32.6	63.0	-2.8	61.4	-1.7	93.1	0.97	0.0	-37.0	58.7	-3.2
1ytf :D	38.2	4.6	78.8	1.14	76.7	10.3	50.0	6.3	32.4	3.8	78.6	1.13	80.0	8.6	46.9	5.2

TAB. 9 – Left : JET with clustering based on conservation and physical-chemical properties (PC). Right : iJET with $i = 10$ where selection of clustered residues is obtained by a consensus of 7 distinguished runs. The proteins listed are all the transients in the Huang benchmark

Homodimers					
pdb code	size	20-39	40-59	60-79	80-98
1bnc :A	433	0	774	136	50
1daa :A	277	854	46	2	0
1dpg :A	485	778	86	2	1
1ecp :A	237	403	158	45	47
1efu :A	364	0	53	783	105
1efu :B	282	120	284	59	7
1frp :A	321	267	206	43	26
1fuq :A	456	161	528	229	18
1gdh :A	320	936	12	0	1
1ges :A	448	576	192	113	30
1glq :A	209	582	25	13	40
1gp1 :A	184	71	47	21	27
1gpm :B	499	5	487	169	32
1hur :A	180	163	400	144	159
1hyh :A	297	860	35	0	1
1ids :A	198	462	405	35	28
1ies :A	174	217	258	29	44
1leh :A	364	22	140	28	3
1mas :A	308	497	11	37	2
1mld :A	313	299	384	40	49
1nhk :L	143	59	642	181	1
1nqv :A	154	349	525	12	1
1oro :A	213	416	167	78	37
1osj :A	345	261	679	0	4
1pky :A	464	478	410	24	30
1poy :1	323	708	92	24	5
1qor :A	326	714	207	18	10
1rah :B	153	63	30	33	8
1scu :D	288	220	279	296	105
1scu :E	388	104	512	119	18
1set :A	202	793	29	0	2
1sft :A	421	717	110	2	3
1tph :1	245	222	542	91	66
1xik :A	340	170	17	46	50
2cst :A	411	403	302	3	30
2eip :A	168	195	240	73	35
2hhm :A	272	855	43	7	18
2pcd :B	200	145	80	6	12
2pcd :N	233	86	71	35	22
2pol :A	366	459	153	46	17
3lad :A	472	312	616	20	14
3mde :A	385	748	177	21	24
6gsv :A	217	463	46	108	13
8cat :A	498	160	677	35	41

Tab. 10 – Size (amino-acids number) and number of sequences retrieved by PSI-BLAST for all homodimer proteins in the Huang database. Number of sequences are considered after filtering and they are reported for each sequence identity class.

Heterodimers					
pdb code	size	20-39	40-59	60-79	80-98
1all :A	160	219	14	10	12
1all :B	161	187	40	3	21
1hcg :A	229	598	51	14	11
1hcg :B	51	320	109	8	2
1luc :A	326	231	40	7	11
1luc :B	320	65	59	3	3
1scu :D	288	220	279	296	105
1scu :E	388	104	512	119	18
1tco :A	352	508	165	44	62
1tco :B	169	691	50	19	29
1tco :C	107	81	690	37	37
1tcr :A	202	354	73	27	2
1tcr :B	236	771	59	113	14
1ubs :A	257	617	75	52	97
1ubs :B	389	5	616	41	172
1wdc :A	64	52	235	19	5
1wdc :B	142	665	141	1	12
1wdc :C	152	621	160	7	2
2pcd :B	200	145	80	6	12
2pcd :N	233	86	71	35	22
8atc :A	310	683	158	40	34
8atc :B	146	63	27	38	7
9atc :A	310	697	151	40	23
9atc :B	146	62	27	36	8

Tab. 11 – Size (amino-acids number) and number of sequences retrieved by PSI-BLAST for all heterodimer proteins in the Huang dataset. Number of sequences are considered after filtering and they are reported for each sequence identity class.

Transients					
pdb code	size	20-39	40-59	60-79	80-98
1apm :E	341	355	295	12	124
1efu :A	364	0	53	783	105
1efu :B	282	120	284	59	7
1g3n :A	293	254	471	25	9
1g3n :B	155	896	1	0	8
1g3n :C	233	269	0	0	5
1got :A	326	184	313	207	12
1got :B	339	556	68	44	88
1got :G	58	143	11	14	6
1k9o :E	223	206	406	141	37
1k9o :I	376	845	9	1	14
1rrp :A	204	718	3	56	69
1rrp :B	134	134	114	27	3
1rrp :C	180	709	0	20	87
1ugh :E	223	78	512	9	13
1ugh :I	82	32	0	0	0
1ytf :A	180	100	43	40	73
1ytf :B	46	18	39	6	1
1ytf :D	100	11	51	2	0

Tab. 12 – Size (amino-acids number) and number of sequences retrieved by PSI-BLAST for all transient proteins in the Huang dataset. Number of sequences are considered after filtering and they are reported for each sequence identity class.
