# Supplementary material for: Joint Evolutionary Trees: A Large-Scale Method To Predict Protein Interfaces Based on Sequence Sampling
Source: PLoS Comput Biol. 2009 Jan 23;5(1):e1000267. doi: 10.1371/journal.pcbi.1000267 (PMC2613531; doi:10.1371/journal.pcbi.1000267)
Supplement: Text S3 — JET and iJET performance on a pool of selected proteins discussed in the article (0.08 MB PDF) [file pcbi.1000267.s003.pdf]

JET and iJET performance on a pool of proteins discussed in the text

Proteins discussed in the text - with clustering																
	**JET on conservation**								**JET on conservation and pc properties**							
PDBCode	Sen	ScSen	PPV	ScPPV	Spe	ScSpe	Acc	ScAcc	Sen	ScSen	PPV	ScPPV	Spe	ScSpe	Acc	ScAcc
1all :A	46.7	18.4	56.8	1.7	81.4	9.6	69.5	12.7	44.4	20.0	62.5	1.8	86.0	10.5	71.8	13.8
1all :B	51.1	23.0	60.5	1.8	83.3	11.5	72.6	15.3	66.7	36.3	73.2	2.2	87.8	18.1	80.7	24.2
1apm :E	52.2	41.2	85.7	4.8	98.1	9.0	89.8	14.8	43.5	33.7	80.0	4.5	97.6	7.4	87.9	12.1
1grn :A	81.8	54.2	45.0	3.0	82.1	9.7	82.1	16.5	68.2	48.2	51.7	3.4	88.6	8.6	85.5	14.6
1grn :B	65.5	47.2	70.4	3.6	93.2	11.6	87.8	18.6	62.1	43.7	66.7	3.4	92.4	10.7	86.4	17.2
1leh :A	3.8	-10.4	2.6	0.3	84.6	-1.1	76.7	-2.0	37.0	14.9	16.7	1.7	79.5	1.6	75.3	3.0
1shc :A	65.0	39.1	29.5	2.5	79.3	5.2	77.6	9.2	70.0	47.1	35.9	3.1	83.3	6.3	81.8	11.1
1ycr :A	58,8	19,7	37,0	1,5	67,3	6,4	65,2	9,7	76,5	30,1	40,6	1,6	63,5	9,8	66,7	14,8
2cjk :A	40.0	27.6	82.4	3.2	97.1	9.5	82.5	14.1	37.1	24.0	72.2	2.8	95.1	8.2	80.3	12.3
2ktq :A	43.5	32.3	45.5	3.9	93.1	4.3	87.3	7.6	39.1	27.4	39.1	3.3	91.9	3.6	85.8	6.4
2pol :A	25.4	10.5	39.0	1.7	88.2	3.1	73.8	4.8	30.2	13.8	42.2	1.8	87.7	4.1	74.5	6.3
2ptc :E	61.9	44.4	46.4	3.5	89.2	6.7	85.6	11.7	71.4	56.4	62.5	4.8	93.5	8.5	90.6	14.8
average	49.6	28.9	50.1	2.6	86.4	7.1	79.2	11.1	**53.4**	**32.5**	**53.3**	**2.9**	**87.1**	**8.0**	**80.4**	**12.3**

TAB. 1 – JET performance on a set of proteins discussed individually in the text. Predictions based on conservation and on both conservation and physical-chemical signals are evaluated. Bold characters indicate best performance. The evaluations that we present correspond to the best performance over the 10 runs used to evaluate iJET in Table 2, where the "best performance" corresponds to the highest PPV and Acc values.

Proteins discussed in the text - with clustering																
	**ET**								**iJET : conservation and pc properties**							
PBDCode	Sen	ScSen	PPV	ScPPV	Spe	ScSpe	Acc	ScAcc	Sen	ScSen	PPV	ScPPV	Spe	ScSpe	Acc	ScAcc
1all :A	42.2	20.1	65.5	1.9	88.4	10.5	72.5	13.8	44.4	20.8	64.5	1.9	87.2	10.9	72.5	14.3
1all :B	40.0	15.6	54.5	1.6	83.3	7.8	68.9	10.4	62.2	32.6	70.0	2.1	86.7	16.3	78.5	21.7
1apm :E	58.7	39.9	56.3	3.1	90.0	8.8	84.4	14.4	43.5	33.3	76.9	4.3	97.1	7.3	87.5	12.0
1grn :A	40.9	23.7	36.0	2.4	87.0	4.2	80.0	7.2	77.3	51.1	44.7	2.9	82.9	9.1	82.1	15.5
1grn :B	34.5	18.8	43.5	2.2	89.0	4.6	78.2	7.4	62.1	43.0	64.3	3.3	91.5	10.6	85.7	17.0
1leh :A	3.7	-15.1	2.0	0.2	79.5	-1.7	72.0	-3.0	3.7	-12.2	2.3	0.2	82.8	-1.3	74.9	-2.4
1shc :A	70.0	50.0	41.2	3.5	86.7	6.7	84.7	11.8	60.0	34.7	27.9	2.4	79.3	4.6	77.1	8.2
1ycr :A	41.2	16.5	41.2	1.7	80.8	5.4	71.0	8.1	70.6	27.1	40.0	1.6	65.4	8.9	66.7	13.4
2cjk :A	54.3	32.4	63.3	2.5	89.2	11.1	80.3	16.5	34.3	21.9	70.6	2.8	95.1	7.5	79.6	11.2
2ktq :A	60.9	41.3	36.4	3.1	85.9	5.5	83.0	9.7	41.3	30.6	45.2	3.9	93.4	4.1	87.3	7.2
2pol :A	46.0	21.7	43.3	1.9	82.1	6.4	73.8	9.9	28.6	12.6	40.9	1.8	87.7	3.7	74.2	5.8
2ptc :E	38.1	28.1	50.0	3.8	94.2	4.2	86.9	7.4	71.4	57.1	65.2	5.0	94.2	8.6	91.3	15.0
average	44.2	24.4	44.4	2.3	86.3	6.1	78.0	9.5	**49.9**	**29.4**	**51.0**	**2.7**	**86.9**	**7.5**	**79.8**	**11.5**

TAB. 2 – iJET is compared to ET on a set of proteins discussed individually in the text. Bold characters indicate best performance.

pdb code	size	20-39	40-59	60-79	80-98
1all :A	160	219	14	10	12
1all :B	161	187	40	3	21
1apm :E	341	355	295	12	124
1grn :A	191	15	520	224	73
1grn :B	197	472	23	3	10
1leh :A	364	22	140	28	3
1shc :A	195	3	7	28	5
1ycr :A	85	0	14	6	11
2cjk :A	167	316	175	5	5
2ktq :A	528	475	139	0	13
2pol :A	366	459	153	46	17
2ptc :E	220	271	352	178	19

Tab. 3 – Size (amino-acids number) and number of sequences retrieved by PSI-BLAST for all proteins illustrated in the article. Number of sequences are considered after filtering and they are reported for each sequence identity class.
