# Supplementary material for: Joint Evolutionary Trees: A Large-Scale Method To Predict Protein Interfaces Based on Sequence Sampling
Source: PLoS Comput Biol. 2009 Jan 23;5(1):e1000267. doi: 10.1371/journal.pcbi.1000267 (PMC2613531; doi:10.1371/journal.pcbi.1000267)
Supplement: Text S4 — JET program information (0.09 MB PDF) [file pcbi.1000267.s004.pdf]

How to run JET and default values of its parameters

Installation note : JET has to be installed with ClustalW and naccess. PSI-BLAST can be installed too but this is not a requirement.

List of default values of parameters stored in the config file used in the system :

PSI-BLAST : the program can be called on the server or on local. To reach similar outputs on the server and on local, we coded a local call to PSI-BLAST with the option -t 2 setting the composition-based score adjustment method conditioned on sequence properties, for improving accuracy in finding true positive matches ; attention, because -t 2 is a PSI-BLAST option and not a JET option).

Notice that a PSI-BLAST threshold = 0.005 (this is a statistical significance threshold to include a sequence in the model used by PSI-BLAST to create the PSSM in the next iteration) is used for both server and local calls.

- gap opening cost = 11
- gap extension =3
- e-value = 10^{-5} for extracting sequences with PSI-BLAST. The automatic adjustment of this default value has been discussed in the text, in case not sufficiently many sequences have been retrieved ;
- number of sequences retrieved = 5000
- database used for PSI-BLAST search = nr
- matrix used in PSI-BLAST to fetch homologues = Blosum62
- number of iterations for PSI-BLAST = 3
- output format = text (corresponding to the local PSI-BLAST option `-T F`)

Software location :

- clustalW location
- naccess location
- psiblast location

Sequence filtering :

- alignments issued with PSI-BLAST are selected based on their length which has to satisfy a min cut-off of 80% and a max cut-off of 110% of the reference sequence length ; this ensures overlapping of all aligned sequences ;
- a min cut-off of 20% and a max cut-off of 98% of sequence identity between retrieved sequences and reference sequence ;

Tree construction :

- alignment matrix = Blosum62. An automatic selection for another matrix (Gonnet and HSDM) under suitable conditions has been discussed in the text.
- msaNumber n fixes the number of alignments (trees) at $n \geq 1$; -1 asks JET to compute itself the best value depending on the number of retrieved sequences.
- seqNumber n fixes the number of sequences aligned for each tree ; -1 asks JET to compute itself the best value depending on the number of retrieved sequences.
- coverage traces : trace levels are computed for a maximum of 95% of the residues of the reference sequence starting from highest values ;

Examples : the options msaNumber and seqNumber have been implemented in such a way that the set of retrieved (and filtered) sequences will be covered (by random selection) in the best way, whenever possible. The automatic handling of trees by JET (corresponding to the value -1) generates at most 50 trees of at most 50 sequences. For instance :

-msaNumber 1 -seqNumber 50 creates 1 tree of 50 sequences.
-msaNumber 3 -seqNumber -1 creates 3 trees with $N/3$ sequences.
-msaNumber -1 -seqNumber 50 creates $N/50$ trees (with a minimum number of 2 trees).
-msaNumber 1 -seqNumber -1 creates 1 tree with all retrieved (and filtered) sequences.
-msaNumber -1 -seqNumber -1 creates $\sqrt{N}$ alignments of $\sqrt{N}$ sequences.

Clustering :

- radius of probe used for accessible surface detection = 1.4Å ;
- minimum percentage of accessible surface for a residue to be considered accessible = 5% ;
- minimum accessible surface for an atom to be considered accessible = $1Å^2$;
- maximum distance between residues to aggregate them in clusters = 5Å ;
- mean coverage of clusters (relative to protein surface), calculated by JET (-1) as a function of surface size using the curve in Figure 4, or provided by the user ;

Calculation of protein interacting sites :

- percentage variation of accessible surface of a residue computed within a protein complex and a single protein has to be higher than 10% for the residue to belong to the interaction site ;
- ligand set to yes (no) to take into account ligands in the PDB database for the evaluation of predicted interactions ;
- homologousPDB set to yes (no) to add interface residues of homologous structures for the evaluation of predicted interactions ;

Command lines and options to exploit JET functioning modes

Command lines :
`java jet.JET [option]`

Mandatory :
-c config-file : file containing all values of parameters discussed above
-i input-file : input pdb file or directory with all input pdb files. These files must match to the pattern pdbCode_chain.pdb
-o output-directory : directory where JET output files will be generated
-p type-of-program {AIJCR} : A to compute accessibility of residues and atoms, I to compute interface residues if the pdb input file is a complex, J to launch JET analysis, C to launch the clustering algorithm, and R to evaluate jet results according to real interface residues (I analysis needed)

Optional :
-l log-file : file containing characteristics of the JET analysis for each protein (pdb code, length of the protein, number of retrieved sequences in identity classes)
-b blast-file : PSI-BLAST input file or directory containing PSI-BLAST input files used by a large scale JET analysis. These files must match to the pattern pdbCode_chain.psiblast
-w pdb_code : pdb code of a structural complex or of a protein that the user wants to analyze. The pdb file corresponding to the pdb code is retrieved on the pdb database site found in the config file
-f fasta-file : fasta input file or directory fasta input files used by the JET analysis. These files must match to the pattern pdbCode_chain.fasta
-m merging-option {T|F} : if "T", several input pdb files with same pdb code are merged in one pdb file. If "F", no merging is done. This option merges pdb files (containing different chains of a complex) and allows calculation of real interfaces ; an example of accepted pdb file names : 1apm_A.pdb et 1apm_B.pdb
-s coverage-threshold $]0.0, 0.5[$: mean coverage of clusters computed by JET
-n nb (1,50) : if $nb > 1$, iJET runs with nb iterations. For $nb = 1$, basic JET is run
-t threshold (1,50) : in iterative mode (see option -n) residues which appear a number of times $\geq$ threshold are selected
-a type-of-analysis {1|2} : 1 for JET analysis based on conservation properties (trace), 2 for JET analysis based on conservation (trace) and physical-chemical properties (pc)
-h : online help
-d accessType {chain|complex} : accessibility computed on a chain (chain) or on a complex (complex) ; in this latter case certain residues in the complex will be inaccessible because belonging to the complex interface ; attention here, because residues at the complex interface are not necessarily inaccessible.
-r retrieving-method {input ;server ;local} : input for input file (assume the use of option -b or -f) ; local for local psiblast analysis (the command to run local psiblast must appear in the config-file) ; server for server psiblast analysis (web address of the psiblast server must appear in the config-file).

-g pdb-results-file (name1,name2, ...) : list of names separated by commas in parenthesis. Names are those of the results columns in the results files {tr ;freq ;pc ;trace ;clusters ;axs ;surfAxs ;percentSurfAxs ;inter ;atomAxs ;atomSurfAxs}. Columns results selected with these names are written in pdb file format (temperature factor column of the pdb file containing value of selected column) and could be viewed in rasmol/VMD/etc. Example : (trace,clusters) if the user wants to have trace and clusters results in pdb format.

JET output.

JET outputs several main files collecting results :

1. JET prediction (<filename>_jet.res). For each residue in the reference sequence S, it lists : residue name r_j, position j in the chain, chain name, trace $d(j)$, trace frequency in the generated trees (number of residues with an average trace value $trace(j) \neq 0$/ number of trees), propensity value, trace*frequency, accessibility (0 if not accessible, 1 if accessible), mixed trace for clustered residues.

If iJET is run instead, with $i \neq 1$, then the file <filename>_jet.res contains some extra columns. For each residue in the reference sequence S, it lists : residue name r_j, position j in the chain, chain name, trace $d(j)$, trace frequency in the generated trees (number of residues with an average trace value $trace(j) \neq 0$/ number of trees), propensity value, trace*frequency for all runs, maximal trace value obtained in all runs, mixed trace for clustered residues calculated for each run, number of runs that the residue appeared in a cluster.

2. NACCESS output (<filename>_axs.res). It is rearranged in a new format : residue name, position in the chain, chain name, whether or not it is an accessible residue, the accessible surface a_j, the fraction of the residue accessible surface.

The file <filename>_atomAxs.res contains information on atomic accessibility. Columns are : atom code, atom position, whether or not it is accessible, the fraction of atom accessible surface.

3. Clusters information (<filename>_clusters.res). It contains : residue name, position, chain, mixed trace value if the residue belongs to a cluster, 0 otherwise. This information is included in <filename>_jet.res.

4. Characteristics of the computation (caracTest.dat) : protein name, chain name, enzyme compounds, names of homologous PDB structures, protein size, number of retrieved sequences after filtering that occur in the four identity classes. This file can be found in the same directory where the program JET is located and collects the history of all PDB files that have been executed.

Example for the protein 1APM, chain E :

```
>1apm :E
EnzymeCompound     ATP ADP GDP PHA GNP ADP
HomologousPDB     1atp 1jbp
size     341
partition     370 ;242 ;12 ;131
```

JET also generates PDB files where the "temperature factor" column is replaced with specific information depending on user defined options to visualize properties such as surface clusters and trace significance with some visualization program like VMD, rasmol or others.
