# Supplementary material for: Joint Evolutionary Trees: A Large-Scale Method To Predict Protein Interfaces Based on Sequence Sampling
Source: PLoS Comput Biol. 2009 Jan 23;5(1):e1000267. doi: 10.1371/journal.pcbi.1000267 (PMC2613531; doi:10.1371/journal.pcbi.1000267)
Supplement: Text S5 — iJET performance on the Kanamori dataset (0.10 MB PDF) [file pcbi.1000267.s005.pdf]

Kanamori dataset - Enzymes												
pdbCode	chain	chain	TP	TN	FP	FN	Sen	Spe	PPV	Acc	Cov	MCC
1a4y	A	B	24	300	28	49	32.9	91.5	46.2	80.8	13	0.28
1acb	E	I	26	154	32	13	66.7	82.8	44.8	80	25.8	0.428
1ava	A	C	6	296	36	65	8.5	89.2	14.3	74.9	10.4	-0.03
1avw	A	B	16	230	25	25	39	90.2	39	83.1	13.9	0.292
1azz	A	D	9	223	39	15	37.5	85.1	18.8	81.1	16.8	0.168
1azz	A	C	18	202	34	31	36.7	85.6	34.6	77.2	18.2	0.218
1bqq	M	T	25	195	28	41	37.9	87.4	47.2	76.1	18.3	0.275
1bvn	P	T	32	289	28	21	60.4	91.2	53.3	86.8	16.2	0.49
1cdk	A	I	17	203	16	15	53.1	92.7	51.5	87.6	13.1	0.452
1clv	A	I	20	277	9	32	38.5	96.9	69	87.9	8.6	0.455
1cse	E	I	26	164	30	9	74.3	84.5	46.4	83	24.5	0.492
1d6r	A	I	21	144	35	18	53.8	80.4	37.5	75.7	25.7	0.301
1dfj	E	I	17	321	22	56	23.3	93.6	43.6	81.2	9.4	0.22
1dhk	A	B	20	364	12	61	24.7	96.8	62.5	84	7	0.322
1dpj	A	B	19	176	10	51	27.1	94.6	65.5	76.2	11.3	0.306
1dtd	A	B	13	184	33	24	35.1	84.8	28.3	77.6	18.1	0.183
1eai	A	C	25	142	47	21	54.3	75.1	34.7	71.1	30.6	0.254
1eja	A	B	19	136	35	17	52.8	79.5	35.2	74.9	26.1	0.279
1f34	A	B	18	245	29	63	22.2	89.4	38.3	74.1	13.2	0.144
1f7z	A	I	23	135	34	10	69.7	79.9	40.4	78.2	28.2	0.407
1fak	H	I	23	144	30	14	62.2	82.8	43.4	79.1	25.1	0.394
1fle	E	I	20	126	44	18	52.6	74.1	31.2	70.2	30.8	0.224
1gl0	E	I	34	136	18	13	72.3	88.3	65.4	84.6	25.9	0.586
1hia	B	I	18	92	48	13	58.1	65.7	27.3	64.3	38.6	0.188
1hx1	A	B	15	290	44	29	34.1	86.8	25.4	80.7	15.6	0.185
1i4o	A	C	9	135	16	9	50	89.4	36	85.2	14.8	0.342
1jiw	I	P	29	334	26	25	53.7	92.8	52.7	87.7	13.3	0.461
1jlt	A	B	29	110	22	48	37.7	83.3	56.9	66.5	24.4	0.236
1jtd	A	B	14	301	27	43	24.6	91.8	34.1	81.8	10.6	0.188
1jtg	A	B	14	240	12	48	22.6	95.2	53.8	80.9	8.3	0.257
1kig	H	I	17	152	30	21	44.7	83.5	36.2	76.8	21.4	0.261
1ppf	E	I	18	139	30	15	54.5	82.2	37.5	77.7	23.8	0.32
1slu	A	B	14	193	36	29	32.6	84.3	28	76.1	18.4	0.159
1stf	E	I	24	143	37	22	52.2	79.4	39.3	73.9	27	0.287
1tbr	H	R	18	153	35	60	23.1	81.4	34	64.3	19.9	0.051
1tmq	A	B	22	307	30	39	36.1	91.1	42.3	82.7	13.1	0.29
1toc	B	R	16	170	31	63	20.3	84.6	34	66.4	16.8	0.058
1ugh	E	I	21	162	12	35	37.5	93.1	63.6	79.6	14.3	0.375
2btc	E	I	26	124	25	13	66.7	83.2	51	79.8	27.1	0.455
2sic	E	I	18	198	30	19	48.6	86.8	37.5	81.5	18.1	0.319
3bth	E	I	21	139	34	13	61.8	80.3	38.2	77.3	26.6	0.353
4htc	H	I	16	125	39	47	25.4	76.2	29.1	62.1	24.2	0.017

TAB. 1 – iJET is evaluated on enzymes of the Kanamori benchmark, for $i = 9$. Columns correspond to chain names, number of true and false positive, number of true and false negatives, evaluation scores, JET coverage, Matthews' correlation coefficient (MCC). TP, TN, FP and FN are defined as the sum of the corresponding values computed on the two chains.

**Kanamori dataset - Inhibitors**												
pdbCode	chain	chain	TP	TN	FP	FN	Sen	Spe	PPV	Acc	Cov	MCC
1a4y	A	B	24	300	28	49	32.9	91.5	46.2	80.8	13	0.28
1ava	A	C	6	296	36	65	8.5	89.2	14.3	74.9	10.4	-0.03
1avw	A	B	16	230	25	25	39	90.2	39	83.1	13.9	0.292
1bqq	M	T	25	195	28	41	37.9	87.4	47.2	76.1	18.3	0.275
1d6r	A	I	21	144	35	18	53.8	80.4	37.5	75.7	25.7	0.301
1df9	B	C	18	151	33	18	50	82.1	35.3	76.8	23.2	0.281
1dfj	E	I	17	321	22	56	23.3	93.6	43.6	81.2	9.4	0.22
1dhk	A	B	20	364	12	61	24.7	96.8	62.5	84	7	0.322
1f7z	A	I	23	135	34	10	69.7	79.9	40.4	78.2	28.2	0.407
1fak	H	I	23	144	30	14	62.2	82.8	43.4	79.1	25.1	0.394
1g73	A	D	9	162	52	6	60	75.7	14.8	74.7	26.6	0.2
1jlt	A	B	29	110	22	48	37.7	83.3	56.9	66.5	24.4	0.236
1k9o	E	I	18	331	54	23	43.9	86	25	81.9	16.9	0.235
1ppf	E	I	18	139	30	15	54.5	82.2	37.5	77.7	23.8	0.32
1sgp	E	I	19	103	38	14	57.6	73	33.3	70.1	32.8	0.256
1tbr	H	R	18	153	35	60	23.1	81.4	34	64.3	19.9	0.051
2sic	E	I	18	198	30	19	48.6	86.8	37.5	81.5	18.1	0.319
3bth	E	I	21	139	34	13	61.8	80.3	38.2	77.3	26.6	0.353

TAB. 2 – iJET (with $i = 9$) is evaluated on inhibitors of the Kanamori dataset.

Kanamori dataset - Signal transduction												
pdbCode	chain	chain	TP	TN	FP	FN	Sen	Spe	PPV	Acc	Cov	MCC
1a2k	A	D	28	170	40	14	66.7	81	41.2	78.6	27	0.4
1agr	A	E	26	304	22	14	65	93.3	54.2	90.2	13.1	0.538
1avz	B	C	18	66	48	7	72	57.9	27.3	60.4	47.5	0.23
1b6c	A	B	16	237	56	24	40	80.9	22.2	76	21.6	0.165
1bdj	A	B	7	109	62	11	38.9	63.7	10.1	61.4	36.5	0.016
1blx	A	B	11	263	53	38	22.4	83.2	17.2	75.1	17.5	0.051
1buh	A	B	12	201	53	19	38.7	79.1	18.5	74.7	22.8	0.132
1c1y	A	B	18	114	44	11	62.1	72.2	29	70.6	33.2	0.263
1c4z	A	D	7	312	49	31	18.4	86.4	12.5	79.9	14	0.041
1cdm	A	B	20	71	18	14	58.8	79.8	52.6	74	30.9	0.374
1cmx	A	B	31	139	34	15	67.4	80.3	47.7	77.6	29.7	0.426
1ds6	A	B	28	204	35	30	48.3	85.4	44.4	78.1	21.2	0.326
1du3	A	D	11	130	39	30	26.8	76.9	22	67.1	23.8	0.035
1du3	A	F	5	126	49	27	15.6	72	9.3	63.3	26.1	-0.102
1eay	A	C	22	89	45	7	75.9	66.4	32.8	68.1	41.1	0.329
1es7	C	D	12	87	45	27	30.8	65.9	21.1	57.9	33.3	-0.03
1es7	A	D	6	102	51	12	33.3	66.7	10.5	63.2	33.3	0
1ev2	A	E	23	198	13	40	36.5	93.8	63.9	80.7	13.1	0.378
1f51	A	E	23	159	47	24	48.9	77.2	32.9	71.9	27.7	0.227
1f5q	A	B	7	299	54	54	11.5	84.7	11.5	73.9	14.7	-0.038
1flt	V	Y	13	105	37	17	43.3	73.9	26	68.6	29.1	0.144
1foe	A	B	34	341	28	33	50.7	92.4	54.8	86	14.2	0.446
1fq1	A	B	17	265	53	21	44.7	83.3	24.3	79.2	19.7	0.218
1fqk	A	B	26	278	26	19	57.8	91.4	50	87.1	14.9	0.463
1g3n	A	C	10	306	46	43	18.9	86.9	17.9	78	13.8	0.057
1g4u	R	S	15	304	41	36	29.4	88.1	26.8	80.6	14.1	0.168
1g4y	B	R	2	110	50	47	4.1	68.8	3.8	53.6	24.9	-0.266
1gcq	B	C	15	34	46	13	53.6	42.5	24.6	45.4	56.5	-0.035
1got	A	B	22	421	43	44	33.3	90.7	33.8	83.6	12.3	0.242
1hcf	B	Y	13	122	36	28	31.7	77.2	26.5	67.8	24.6	0.084
1hcf	A	Y	6	143	49	11	35.3	74.5	10.9	71.3	26.3	0.061
1he1	A	C	26	160	23	24	52	87.4	53.1	79.8	21	0.397
1he8	A	B	10	570	62	22	31.2	90.2	13.9	87.3	10.8	0.148
1i2m	A	B	16	283	32	53	23.2	89.8	33.3	77.9	12.5	0.151
1i4d	A	D	11	237	29	24	31.4	89.1	27.5	82.4	13.3	0.194
1ib1	A	E	30	220	36	37	44.8	85.9	45.5	77.4	20.4	0.309
1ibr	A	B	14	335	41	69	16.9	89.1	25.5	76	12	0.071
1jdp	A	H	0	246	8	20	0	96.9	0	89.8	2.9	-0.049
1jma	A	B	2	231	20	37	5.1	92	9.1	80.3	7.6	-0.037
1k90	A	D	22	366	26	102	17.7	93.4	45.8	75.2	9.3	0.163
1lfd	A	B	19	120	47	10	65.5	71.9	28.8	70.9	33.7	0.281
1lfd	B	C	11	119	58	7	61.1	67.2	15.9	66.7	35.4	0.172
1qav	A	B	16	84	49	26	38.1	63.2	24.6	57.1	37.1	0.011
1qbk	B	C	18	709	45	88	17	94	28.6	84.5	7.3	0.139
1qmz	A	B	12	295	40	61	16.4	88.1	23.1	75.2	12.7	0.052
1rrp	A	B	28	128	34	82	25.5	79	45.2	57.4	22.8	0.052
1tnr	A	R	8	169	45	29	21.6	79	15.1	70.5	21.1	0.005
1tx4	A	B	29	184	36	23	55.8	83.6	44.6	78.3	23.9	0.363
1vrk	A	B	20	82	20	18	52.6	80.4	50	72.9	28.6	0.325
1wq1	G	R	40	259	23	27	59.7	91.8	63.5	85.7	18.1	0.528
1www	V	Y	11	117	38	26	29.7	75.5	22.4	66.7	25.5	0.047
1www	W	Y	7	136	39	13	35	77.7	15.2	73.3	23.6	0.091
1zbd	A	B	22	152	37	30	42.3	80.4	37.3	72.2	24.5	0.217

TAB. 3 – iJET (with $i = 9$) is evaluated on signal transduction proteins of the Kanamori dataset.

Kanamori dataset - Antibodies												
pdbCode	chain	chain	TP	TN	FP	FN	Sen	Spe	PPV	Acc	Cov	MCC
1a2y	B	C	3	110	63	20	13	63.6	4.5	57.7	33.7	-0.159
1a2y	A	C	2	115	57	14	12.5	66.9	3.4	62.2	31.4	-0.124
1bgx	H	T	10	651	81	76	11.6	88.9	11	80.8	11.1	0.005
1bgx	L	T	7	679	73	60	10.4	90.3	8.8	83.8	9.8	0.007
1bj1	H	W	0	166	70	32	0	70.3	0	61.9	26.1	-0.219
1bzq	A	L	10	123	40	47	17.5	75.5	20	60.5	22.7	-0.073
1dee	D	G	14	140	61	14	50	69.7	18.7	67.2	32.8	0.137
1dqj	B	C	3	198	47	20	13	80.8	6	75	18.7	-0.044
1dqj	A	C	0	200	52	21	0	79.4	0	73.3	19	-0.14
1e6j	H	P	1	280	55	25	3.8	83.6	1.8	77.8	15.5	-0.09
1egj	A	H	0	203	46	18	0	81.5	0	76	17.2	-0.123
1eo8	A	H	3	316	64	30	9.1	83.2	4.5	77.2	16.2	-0.057
1fbi	H	X	8	200	61	24	25	76.6	11.6	71	23.5	0.012
1fc2	C	D	7	148	39	21	25	79.1	15.2	72.1	21.4	0.034
1fe8	A	H	0	221	71	22	0	75.7	0	70.4	22.6	-0.148
1fe8	A	L	0	232	61	26	0	79.2	0	72.7	19.1	-0.145
1fj1	B	F	0	351	24	21	0	93.6	0	88.6	6.1	-0.06
1fj1	A	F	0	342	58	26	0	85.5	0	80.3	13.6	-0.101
1fns	A	H	0	224	56	22	0	80	0	74.2	18.5	-0.134
1fsk	A	C	8	241	50	21	27.6	82.8	13.8	77.8	18.1	0.078
1g9m	G	H	5	335	60	20	20	84.8	7.7	81	15.5	0.031
1jhl	A	H	1	103	67	19	5	60.6	1.5	54.7	35.8	-0.22
1jps	H	T	0	262	39	36	0	87	0	77.7	11.6	-0.125
1jtp	A	L	11	111	56	24	31.4	66.5	16.4	60.4	33.2	-0.017
1mlc	B	E	2	211	48	23	8	81.5	4	75	17.6	-0.078
1ncc	L	N	0	363	61	25	0	85.6	0	80.8	13.6	-0.096
1ncc	H	N	1	346	46	27	3.6	88.3	2.1	82.6	11.2	-0.065
1osp	L	O	0	344	38	18	0	90.1	0	86	9.5	-0.07
1osp	H	O	0	331	47	21	0	87.6	0	83	11.8	-0.086
1qkz	A	H	8	140	57	19	29.6	71.1	12.3	66.1	29	0.005
1t83	B	C	4	238	49	17	19	82.9	7.5	78.6	17.2	0.013
1wej	F	L	1	202	52	13	7.1	79.5	1.9	75.7	19.8	-0.074
1wej	F	H	2	204	51	15	11.8	80	3.8	75.7	19.5	-0.05
2hmi	B	D	0	475	31	24	0	93.9	0	89.6	5.8	-0.054
2jel	H	P	2	167	52	23	8	76.3	3.7	69.3	22.1	-0.115
2vir	B	C	0	289	59	33	0	83	0	75.9	15.5	-0.132

TAB. 4 – iJET (with $i = 9$) is evaluated on antibodies of the Kanamori dataset.

Kanamori dataset - Antigens												
pdbCode	chain	chain	TP	TN	FP	FN	Sen	Spe	PPV	Acc	Cov	MCC
1adq	A	H	1	261	66	22	4.3	79.8	1.5	74.9	19.1	-0.1
1bgx	H	T	10	651	81	76	11.6	88.9	11	80.8	11.1	0.005
1bgx	L	T	7	679	73	60	10.4	90.3	8.8	83.8	9.8	0.007
1bj1	H	W	0	166	70	32	0	70.3	0	61.9	26.1	-0.219
1bzq	A	L	10	123	40	47	17.5	75.5	20	60.5	22.7	-0.073
1dqj	B	C	3	198	47	20	13	80.8	6	75	18.7	-0.044
1dqj	A	C	0	200	52	21	0	79.4	0	73.3	19	-0.14
1dzb	A	X	8	192	35	32	20	84.6	18.6	74.9	16.1	0.044
1e0o	A	B	19	205	23	22	46.3	89.9	45.2	83.3	15.6	0.359
1e6j	H	P	1	280	55	25	3.8	83.6	1.8	77.8	15.5	-0.09
1e96	A	B	14	199	39	12	53.8	83.6	26.4	80.7	20.1	0.279
1eo8	A	H	3	316	64	30	9.1	83.2	4.5	77.2	16.2	-0.057
1fbi	H	X	8	200	61	24	25	76.6	11.6	71	23.5	0.012
1fe8	A	L	0	232	61	26	0	79.2	0	72.7	19.1	-0.145
1fns	A	H	0	224	56	22	0	80	0	74.2	18.5	-0.134
1fsk	A	C	8	241	50	21	27.6	82.8	13.8	77.8	18.1	0.078
1g9m	G	H	5	335	60	20	20	84.8	7.7	81	15.5	0.031
1g9m	C	G	3	293	38	51	5.6	88.5	7.3	76.9	10.6	-0.067
1hez	A	E	13	153	47	18	41.9	76.5	21.7	71.9	26	0.143
1jhl	A	H	1	103	67	19	5	60.6	1.5	54.7	35.8	-0.22
1jtp	A	L	11	111	56	24	31.4	66.5	16.4	60.4	33.2	-0.017
1mlc	B	E	2	211	48	23	8	81.5	4	75	17.6	-0.078
1mlc	A	E	1	215	51	13	7.1	80.8	1.9	77.1	18.6	-0.067
1ncc	L	N	0	363	61	25	0	85.6	0	80.8	13.6	-0.096
1ncc	H	N	1	346	46	27	3.6	88.3	2.1	82.6	11.2	-0.065
1t83	B	C	4	238	49	17	19	82.9	7.5	78.6	17.2	0.013
1t83	A	C	0	233	54	22	0	81.2	0	75.4	17.5	-0.127
1wej	F	H	2	204	51	15	11.8	80	3.8	75.7	19.5	-0.05
2jel	H	P	2	167	52	23	8	76.3	3.7	69.3	22.1	-0.115
2vir	B	C	0	289	59	33	0	83	0	75.9	15.5	-0.132

TAB. 5 – iJET (with $i = 9$) is evaluated on antigens of the Kanamori dataset.

Kanamori dataset - Others												
pdbCode	chain	chain	TP	TN	FP	FN	Sen	Spe	PPV	Acc	Cov	MCC
1aip	A	D	7	398	35	17	29.2	91.9	16.7	88.6	9.2	0.163
1aip	A	C	10	372	37	41	19.6	91	21.3	83	10.2	0.109
1ak4	A	D	11	154	57	16	40.7	73	16.2	69.3	28.6	0.096
1aro	L	P	7	577	66	42	14.3	89.7	9.6	84.4	10.5	0.034
1atn	A	D	2	360	46	38	5	88.7	4.2	81.2	10.8	-0.058
1axi	A	B	15	207	30	43	25.9	87.3	33.3	75.3	15.3	0.146
1b41	A	B	14	284	59	35	28.6	82.8	19.2	76	18.6	0.097
1bml	A	C	4	313	52	89	4.3	85.8	7.1	69.2	12.2	-0.122
1bp3	A	B	18	228	25	44	29	90.1	41.9	78.1	13.7	0.222
1cd9	A	B	0	225	44	34	0	83.6	0	74.3	14.5	-0.147
1cic	B	C	0	268	66	20	0	80.2	0	75.7	18.6	-0.117
1d5m	A	C	4	253	32	36	10	88.8	11.1	79.1	11.1	-0.013
1dkg	A	D	9	325	55	38	19.1	85.5	14.1	78.2	15	0.041
1dx5	I	M	8	199	51	24	25	79.6	13.6	73.4	20.9	0.036
1ebd	B	C	7	344	14	19	26.9	96.1	33.3	91.4	5.5	0.254
1efu	A	B	26	401	21	56	31.7	95	55.3	84.7	9.3	0.339
1f60	A	B	36	280	31	47	43.4	90	53.7	80.2	17	0.363
1fak	H	T	5	228	53	19	20.8	81.1	8.6	76.4	19	0.014
1fak	L	T	17	159	41	35	32.7	79.5	29.3	69.8	23	0.117
1gh6	A	B	2	263	38	34	5.6	87.4	5	78.6	11.9	-0.068
1hyr	B	C	0	262	45	28	0	85.3	0	78.2	13.4	-0.119
1hyr	A	C	0	251	45	33	0	84.8	0	76.3	13.7	-0.133
1i1r	A	B	1	303	38	28	3.4	88.9	2.6	82.2	10.5	-0.067
1i5k	A	C	8	47	18	16	33.3	72.3	30.8	61.8	29.2	0.055
1i7w	A	B	34	274	3	64	34.7	98.9	91.9	82.1	9.9	0.495
1icf	A	I	18	120	46	28	39.1	72.3	28.1	65.1	30.2	0.103
1im3	A	D	5	238	32	31	13.9	88.1	13.5	79.4	12.1	0.02
1im9	A	D	0	317	43	30	0	88.1	0	81.3	11	-0.102
1ira	X	Y	11	251	32	77	12.5	88.7	25.6	70.6	11.6	0.016
1itb	A	B	6	245	44	84	6.7	84.8	12	66.2	13.2	-0.108
1j7v	L	R	10	235	40	25	28.6	85.5	20	79	16.1	0.121
1kac	A	B	21	157	47	17	55.3	77	30.9	73.6	28.1	0.261
1kcg	B	C	4	156	52	15	21.1	75	7.1	70.5	24.7	-0.025
1kcg	A	C	2	145	55	24	7.7	72.5	3.5	65	25.2	-0.146
1lpb	A	B	7	289	51	32	17.9	85	12.1	78.1	15.3	0.025
1nrn	H	R	12	121	19	8	60	86.4	38.7	83.1	19.4	0.388
1qo3	A	C	1	250	68	25	3.8	78.6	1.4	73	20.1	-0.116
1sbb	A	B	0	298	44	28	0	87.1	0	80.5	11.9	-0.105
1t7p	A	B	18	504	36	24	42.9	93.3	33.3	89.7	9.3	0.323
1ycs	A	B	15	229	36	20	42.9	86.4	29.4	81.3	17	0.25
2pcc	A	B	7	232	60	23	23.3	79.5	10.4	74.2	20.8	0.02
3c98	A	B	9	457	38	95	8.7	92.3	19.1	77.8	7.8	0.014

TAB. 6 – iJET (with $i = 9$) is evaluated on a list of non-classified proteins (others) of the Kanamori dataset.
